# Interrelating differences in structural and functional connectivity in the older adult's brain

**DOI:** 10.1002/hbm.26030

**Published:** 2022-08-02

**Authors:** Johanna Stumme, Camilla Krämer, Tatiana Miller, Jan Schreiber, Svenja Caspers, Christiane Jockwitz

**Affiliations:** ^1^ Institute of Neuroscience and Medicine (INM‐1), Research Centre Jülich Jülich Germany; ^2^ Institute for Anatomy I, Medical Faculty & University Hospital Düsseldorf Heinrich Heine University Düsseldorf Düsseldorf Germany

**Keywords:** aging, cognitive performance, functional connectivity, multivariate analyses, structural connectivity

## Abstract

In the normal aging process, the functional connectome restructures and shows a shift from more segregated to more integrated brain networks, which manifests itself in highly different cognitive performances in older adults. Underpinnings of this reorganization are not fully understood, but may be related to age‐related differences in structural connectivity, the underlying scaffold for information exchange between regions. The structure–function relationship might be a promising factor to understand the neurobiological sources of interindividual cognitive variability, but remain unclear in older adults. Here, we used diffusion weighted and resting‐state functional magnetic resonance imaging as well as cognitive performance data of 573 older subjects from the 1000BRAINS cohort (55–85 years, 287 males) and performed a partial least square regression on 400 regional functional and structural connectivity (FC and SC, respectively) estimates comprising seven resting‐state networks. Our aim was to identify FC and SC patterns that are, together with cognitive performance, characteristic of the older adults aging process. Results revealed three different aging profiles prevalent in older adults. FC was found to behave differently depending on the severity of age‐related SC deteriorations. A functionally highly interconnected system is associated with a structural connectome that shows only minor age‐related decreases. Because this connectivity profile was associated with the most severe age‐related cognitive decline, a more interconnected FC system in older adults points to a process of dedifferentiation. Thus, functional network integration appears to increase primarily when SC begins to decline, but this does not appear to mitigate the decline in cognitive performance.

## Graphical abstract

Aging is accompanied by a functional reorganization which manifests itself in cognitive performance differences, especially in older adults. Sources of functional reorganization are not fully understood but have been associated with structural connectivity differences. Based on a large cohort of older adults we show that functional connectivity behaves in relation to age‐related structural connectivity differences and further, that a functional dedifferentiated system, associated with minor structural connectivity decreases, is nonbeneficial in terms of cognitive performance maintenance.

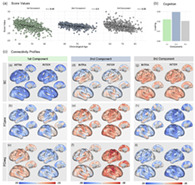

## 1 INTRODUCTION

Age‐related decreases in cognitive performance have been associated with numerous neural substrates (Hedden et al., 2016; MacDonald & Pike, 2021; Whalley et al., 2004) including age‐related differences in the brain network configuration (for reviews, see, Damoiseaux, 2017; Salat, 2011; Sporns, 2013; Wig, 2017; Zuo et al., 2017). Brain networks comprise sets of brain regions (nodes) and their connections (edges) which together are associated with solving specific behavioral tasks (Schaefer et al., 2018; Smith et al., 2009; Yeo et al., 2011). Thereby, brain regions belonging to the same network are more highly connected (intra‐network) as compared to regions outside its related network (inter‐network). The entirety of all connected regions within and across networks forms the whole‐brain connectome (Bullmore & Sporns, 2009, 2012; Fornito, 2016; Fornito et al., 2013) that seems to be subject to age‐related reorganization in terms of both, functional as well as structural connectivity (FC and SC).

In young adults, an efficient functional network configuration, which is associated with high cognitive performance, is characterized by a balance between connections of regions belonging to the same and other networks (Bullmore & Sporns, 2012; Sadaghiani et al., 2015; Sporns, 2013; Wig, 2017). With increasing age, however, this segregated and specialized network configuration decomposes, showing a shift towards a higher network integration, that is, decreasing intra‐network FC and increasing inter‐network FC (Betzel et al., 2014; Cao et al., 2014; Chan et al., 2014; Ferreira et al., 2016; He et al., 2020; Mowinckel et al., 2012; Tsvetanov et al., 2016; Varangis et al., 2019). Across the adult lifespan, intra‐network FC decreases predominantly pertain to higher‐order networks, for example, the default mode network (DMN) and frontoparietal network. In contrast, primary processing networks, for example, the sensorimotor (SMN) and visual network (VN) remain rather stable (Betzel et al., 2014; Chan et al., 2014; Ferreira et al., 2016; Geerligs et al., 2015; Grady et al., 2016; Jockwitz & Caspers, 2021; Mowinckel et al., 2012; Siman‐Tov et al., 2016; Spreng et al., 2016; Varangis et al., 2019). In older adults, though, differences in primary processing networks become highly apparent with age‐related intra‐network FC decreases together with FC increases with higher order networks (Edde et al., 2021; Perry et al., 2017; Stumme et al., 2020; Zonneveld et al., 2019).

The origins of these age‐related FC changes, from segregated toward integrated networks, are not fully understood and their effect is ambiguously interpreted. On one hand, the functional recruitment of additional brain networks is understood as a compensation strategy in older adults, in which age‐related decreases in intra‐network FC may be compensated by functional adaptations (Cabeza et al., 2002; Marstaller et al., 2015; Pistono et al., 2021; Reuter‐Lorenz & Cappell, 2008) to countervail cognitive performance decline (Bartres‐Faz & Arenaza‐Urquijo, 2011; Spreng & Turner, 2019; Stern, 2002, 2009). On the other hand, age‐related shifts toward increasing inter‐network connectivity are thought to result from longer latencies in dynamic functional states, that is, a decreased variance in functional dynamics across time (Battaglia et al., 2020; Naik et al., 2017). A functional system with less variance in functional dynamics is understood as a dedifferentiated system in which the ability to recruit specialized neural mechanisms and to switch between brain states is reduced, followed by a cognitive decline (Chan et al., 2014, 2017; Colcombe et al., 2005; Goh, 2011; Nashiro et al., 2017; Park et al., 2004). In fact, the origin of these age‐related functional reorganizations and the underlying mechanism, being it compensation or dedifferentiation, still remains unclear. To further elucidate this, the additional analysis of SC could be helpful as it provides the structural framework for FC.

SC was found to decrease across aging, spanning the whole brain but with a particular vulnerability of the frontal lobe (Antonenko & Floel, 2014; Betzel et al., 2014; Gunning‐Dixon et al., 2009; Puxeddu et al., 2020; Westlye et al., 2010; Zhao et al., 2015; Zuo et al., 2017). In a recent study of older adults, age‐related disruption of the structural connectome was found to impair both network segregation and network integration (Li et al., 2020). As such, age‐related alterations in SC may relate to the disrupted balance between network integration and segregation in FC. So far, the interrelation between SC and FC and their differences across aging are still a matter of debate. While there exist many studies characterizing age‐related differences in terms of functional and structural networks in isolation (for reviews, see, Damoiseaux, 2017; Jockwitz & Caspers, 2021; Wig, 2017; Zuo et al., 2017), there are fewer studies that have jointly examined FC and SC in the aging process (for review, see, Lynn & Bassett, 2019; Straathof et al., 2019). Results on the direct relation between FC and SC in terms of age‐related differences appear mixed. On the one hand, FC and SC were found to change mostly independently across the lifespan (Fjell et al., 2017; Hirsiger et al., 2016; Tsang et al., 2017) as well as in older adults (Hirsiger et al., 2016) indicating that SC only weakly influences or constricts age‐related differences in FC. On the other hand, studies suggest that SC and FC are interrelated, and that during adolescence changes in the structural connectome are associated with the development and specialization of functional systems (Baum et al., 2020). Across the lifespan, Zimmermann et al. (2016) found increasing age to be accompanied by a greater coupling between SC and FC, which may be explained by the fact that more strongly integrated functional systems (as present in older adults) were found to be more strongly rely on existing structural pathways (Fukushima et al., 2018). With regards to cognitive performance, Davis et al. (2012) found that functional overactivation in older adults during task execution, for example, in contralateral regions, depends on the integrity of the interhemispheric SC. This suggests that functional restructuring in older adults is related to SC in the sense that the ability to recruit additional brain areas, that is, to meet increasing task demands, is mediated by the underlying SC. To date, however, no study has looked at the relationship between whole‐brain structural, functional connectivity, and cognition in older adults. By analyzing this triad, we aim to shed light on the possible causes of the functional shift in older adults.

Specifically, we took advantage of a large sample of older adults from the 1000BRAINS study to investigate SC and FC differences that are jointly age‐characteristic and related to cognition. To investigate this, we used partial least squares regression (PLSR) (Mevik et al., 2018), which, in contrast to univariate approaches, has the great potential to effectively deal with high dimensional data. PLSR capitalizes on the potential to detect interrelations between various predictor variables such as region‐wise connectivity estimates (comprising all networks) and cognition (Chen et al., 2019; Krishnan et al., 2011; McIntosh & Lobaugh, 2004; Yoo et al., 2018). PLSR decomposes predictor variables (cognition, SC, and FC estimates) into smaller sets of independent components, that is, aging profiles, that are maximally correlated with age. These aging profiles unveil region‐wise estimates of SC and FC that are together related to cognition and particularly age‐characteristic. As we investigate an older adult sample (55–85 years), we assume low FC of primary processing networks together with high FC between higher‐order networks to be age‐characteristic. With respect to SC, we hypothesize that older adults have lower connectivity overall, particularly in regions of the frontal lobe. How region‐wise age‐related SC and FC differences are interrelated, tough, is uncertain and analyzed from an explanatory, holistic perspective.

## 2 METHODS

### 2.1 Subjects

The subjects of the current study are drawn from 1000BRAINS (Caspers et al., 2014), a large longitudinal population‐based cohort study investigating the interindividual variability in brain structure, function, and connectivity and its relations to behavioral, environmental, and genetic factors. Subjects included in 1000BRAINS were recruited from the 10‐year follow‐up of the epidemiological population‐based Heinz Nixdorf Recall Study, a study investigating risk factors for atherosclerosis, cardiovascular disease, cardiac infarction, and cardiac death (Schmermund et al., 2002). 1000BRAINS aims at characterizing the aging process at the level of the general population, therefore no exclusion criteria other than eligibility for MR measurements (Caspers et al., 2014) were applied. 1000BRAINS comprises 969 older adults aged between 55 and 88 years of one measurement time point, as relevant for the current cross‐sectional study design. From the initial sample, 114 participants had to be excluded due to preprocessing failure caused by artifacts in structural T1 scans, problems during normalization procedure, or insufficient AROMA‐denoising (*n* = 98). Subsequently, functional data were quality checked and excluded in cases of insufficient quality (*n* = 16, see Section 2.2.2 for description of quality control). Of these 855 participants, 720 subjects also had diffusion‐weighted images available, from which another 69 were excluded after quality control of the diffusion‐weighted images (see Section 2.2.1 for the description of quality control). Finally, participants with missing information on education (*n* = 1), the dementia screening test (*n* = 13, DemTect; Kalbe et al., 2004), or those with indication for potential cognitive impairment (score of eight or lower, *n* = 1) according to the dementia screening test were excluded. After the exclusion of participants with more than three missing values in the cognitive performance tests as well as outliers (mean ± 3 * standard deviation[SD]), the final study sample comprises *n* = 573 subjects (Table 1). All subjects gave written informed consent prior to inclusion in 1000BRAINS. The study protocol of 1000BRAINS was approved by the Ethics Committee of the University of Essen, Germany. Due to local regulations of data acquisition and usage, data of 1000BRAINS are available upon request from the responsible principal investigator.

**TABLE 1:** Descriptives of the study sample

	*n*, proportion in %	Age in years			Education		
		Mean (SD)	Min	Max	Mean (SD)	Min	Max
All	573, 100%	66.9 (6.7)	55.1	85.4	6.5 (1.9)	3	10
Male	287, 50.1%	67.6 (7.0)	55.1	85.4	7.1 (1.9)	3	10
Female	286, 49.9%	66.2 (6.4)	55.2	85.4	6.0 (1.9)	3	10

### 2.2 Imaging

Magnetic resonance imaging was performed using a 3T Siemens Tim‐TRIO MR scanner with a 32‐channel head coil (Erlangen, Germany). For the investigation of SC and FC, different sequence images were included in the current study (see Caspers et al. (2014) for a detailed description of the 1000BRAINS study protocol): For surface reconstruction, a three‐dimensional high‐resolution T1 weighted magnetization‐prepared rapid acquisition gradient‐echo (MPRAGE) anatomical scan was acquired [176 slices, slice thickness 1 mm, repetition time (TR) = 2250 ms, echo time (TE) = 3.03 ms, field of view (FoV) = 256 × 256 mm^2^, flip angle = 9°, voxel resolution 1 × 1 × 1 mm^3^]. For structural connectivity analyses, high‐angular resolution diffusion imaging (HARDI) data with the following parameters were used: (1) 120 directions dataset; EPI, TR = 8 s, TE = 112 ms, 13 b0‐images (interleaved), 120 images with b = 2700 s/mm^2^, voxel resolution = 2.4 × 2.4 × 2.4 mm^3^; (2) 60 direction subset (out of 120 direction dataset); EPI, TR = 6.3 s, TE = 81 ms, 7 b0‐images (interleaved), 60 images with b = 1000 s/mm^2^, voxel resolution = 2.4 × 2.4 × 2.4 mm^3^. For functional connectivity analysis, resting‐state functional MRI data were acquired as a blood‐oxygen level‐dependent (BOLD) gradient‐echo planar imaging (EPI) sequence with 36 transversally oriented slices (slice thickness 3.1 mm, TR = 2200 ms, TE = 30 ms, FoV = 200 × 200 mm^2^, voxel resolution 3.1 × 3.1 × 3.1 mm^3^) was used, lasting for ~11 min and producing 300 volumes. During RS image acquisition, participants were instructed to keep their eyes closed, be relaxed, let their mind wander, and to not fall asleep. The latter was assured by postscan debriefing.

#### 2.2.1 Structural image processing

For each participant, tissue probability maps (TPM) for grey matter (GM), white matter (WM) as well as corticospinal fluid (CSF) were computed from T1 data using the Computational Anatomy Toolbox (CAT12; Gaser & Dahnke, 2016) implemented in SPM12 (Ashburner, 2009; for a listing of software used see Table S1). To optimally extract the brain from the T1 data, brain masks were used created by superimposing the three probability maps and thresholding them at 0.5 (small enclosed holes were filled). Using the FSL toolbox (FMRIB Software Library: http://www.fmrib.ox.ac.uk/fsl; Jenkinson et al., 2012), the T1 brain image was bias field corrected, rigidly aligned to MNI152 template space, and resampled to 1.25 mm isotropic voxel size. These scans were then used as coregistration image for the subsequent alignment of the similarly resampled diffusion data (see below) to the MNI152 template [in accordance with standard pipelines as used in, e.g., the human connectome project (www.humanconnectomeproject.org) or the UK Biobank (www.ukbiobank.ac.uk)]. Diffusion MRI data (dMRI) were corrected for eddy current and motion artifacts including interpolation of slices with signal dropouts (Andersson et al., 2016; Andersson & Sotiropoulos, 2016). Visual quality control was performed to check for ghosting, remaining signal dropouts, or very noisy data. Suboptimal volumes or datasets were removed from further analyses (*n* = 69). For dMRI‐T1 alignment, the first b0 images from each dMRI data with b1000 and b2700 were extracted and rigidly aligned to T1 dataset using mutual information as a cost function (Wells et al., 1996). Based on the corresponding transforms, all dMRI data were registered to the individual T1 space, separately for the two b‐values. The realignment implicitly resampled the data to 1.25 mm and b‐vectors were rotated according to the transformations. To account for susceptibility artifacts and optimize image registration, we computed Anisotropic Power Maps (APM; Dell'Acqua et al., 2014) from the b2700 dMRI data. Since the APM contrast is very similar to the T1 image, they provide an optimal basis for image registration. Accordingly, APMs were used to compute the nonlinear transformation from diffusion to anatomical space additionally taking EPI‐induced distortions into account using ANTs (https://stnava.github.io/ANTs/). The derived nonlinear transformations were then used to transform the TPMs to diffusion space. Finally, the two datasets with b1000 and b2700 were merged into one single file and corrected for different echo times. This correction was computed by a voxel‐wise multiplication of the b2700 data with the ratio of the nondiffusion‐weighted data, respectively, for the two datasets. Subsequently, local modeling and probabilistic streamline tractography were performed using the MRtrix software package (Tournier et al., 2012) version 0.3.15. The constrained spherical deconvolution (CSD) local model was computed using multi‐tissue CSD of multi‐shell data (Jeurissen et al., 2014) using all shells and a maximal spherical harmonic order of 8. Ten million streamlines were computed with dynamic seeding in the grey‐white matter interface for every subject using the probabilistic iFOD2 algorithm with a maximal length of 250 mm and a cut‐off value of 0.06.

#### 2.2.2 Functional image processing

Functional image preprocessing was performed using the FSL toolbox (FMRIB Software Library: http://www.fmrib.ox.ac.uk/fsl; Jenkinson et al., 2012). For each participant, the first four echo‐planar imaging (EPI) volumes were discarded. Using a two‐pass procedure, all functional images were corrected for head movement using rigid‐body registration. First, all volumes were aligned to the first image on which a mean image was created serving as the basis to which secondly, all volumes were aligned. To identify and remove motion‐related independent components from functional MRI data, ICA‐based Automatic Removal Of Motion Artifacts (ICA‐AROMA; Pruim et al., 2015) was applied. According to current suggestions for minimizing the relationship between motion and resting‐state FC (Burgess et al., 2016; Ciric et al., 2017; Parkes et al., 2018), AROMA was combined with global signal regression in the current study. Finally, all resting‐state fMRI images were bandpass filtered (0.01–0.1 Hz) and registered to the standard space template (MNI152) using the unified segmentation approach (Ashburner & Friston, 2005). This was preferred to normalization based on T1 weighted images as previous studies indicated increased registration accuracies (Calhoun et al., 2017; Dohmatob et al., 2018). With AROMA particularly focusing on the correction of intensity artifacts induced by head motion, we further on took advantage of an established algorithm by Afyouni and Nichols (2018) to check for each participant's volume‐wise severe intensity dropouts by generating *p* values for spikes (DVARS) on the already preprocessed functional data. In the current study, volumes with corrupted spikes are indicated and participants for which more than 10% of the 300 volumes (Stumme et al., 2020) were detected as dropouts were excluded from further analyses (*n* = 8). Further, based on the preprocessed mean AROMA functional data, we checked for potential misalignments by performing the “check sample homogeneity using standard deviation across sample” function provided by the CAT12 toolbox (Gaser & Dahnke, 2016) and excluded participants for which the individual image did not align to the MNI152 template (>2 SD, *n* = 8).

### 2.3 Connectivity analyses

To analyze FC and SC data, we parcellated the whole brain into 400 different regions comprising seven networks [visual (VN), sensorimotor (SMN), limbic (LN), frontoparietal (FPN), default mode (DMN), dorsal (DAN), and ventral attention network (VAN)], as defined in Yeo et al. (2011) using the predefined cortical parcellation of Schaefer et al. (2018). This was done according to recent studies, which found a resolution of 300–600 nodes to be optimal for functional (Schaefer et al., 2018) and structural analyses (Varikuti et al., 2018).

In terms of FC, mean time‐series spanning 296 time points (first four of in total 300 volumes were discarded) were extracted node‐wise from the preprocessed resting‐state fMRI data [fslmeants (Smith et al., 2004)] averaging the timeseries of all voxels corresponding to that node. FC between nodes was estimated using Pearson's product–moment correlation of the respective average BOLD time series resulting in a symmetric 400 × 400 matrix, with each entry (i.e., edge) representing a Pearson's correlation coefficient between the respective nodes. To minimize the number of edges caused by noise, we included the statistical significance of each correlation coefficient as an additional preprocessing step. Therefore, the observed time‐series were randomized by taking its Fourier transform, scrambling its phase, and then inverting the transform (Stumme et al., 2020; Zalesky et al., 2012). This procedure was repeated 1000 times and followed by a permutation test (nonsignificant edges at *p* ≥ .05 were set to zero). The adjacency matrix was then transformed into *z*‐scores by applying a Fishers *r*‐to‐*z* transformation. Integrating both, positive as well as negative weights into the estimation of strength values leads to a mutual suppression by canceling each other out. Therefore, we separated the FC matrices, with one containing only positive correlations (FC_pos_) and the second containing only the absolute values of negative correlations (FC_neg_), with the other values set to zero in each case.

Regarding SC, the parcellation template was first warped to individual diffusion space by combining the nonlinear warps of the spatial T1 registration to MNI152 template and the distortion correction with the APMs. Since streamlines are generated seeding from the grey‐white matter interface and the predefined parcellation scheme only covers cortical grey matter, we expanded the template adding voxels towards the grey‐white matter boundary so that all regions also include the seeding points. To increase the biological accuracy of SC, we converted streamline counts between each pair of nodes into weighting factors using a cross‐sectional area multiplier (SIFT‐2; Smith et al., 2015). Finally, the derived 400 × 400 matrix was log10 transformed.

Each of the SC, FC_pos_ as well as FC_neg_ whole brain connectomes (i.e., 400 × 400 connectivity matrices) were then transformed into a triangular matrix (diagonal set to NaN) as only unidirectional information of edges was used. Based on the three different matrices, we calculated two different parameters for each node:

- Intra‐network connectivity estimate comprising the sum of weights (i.e., connectivity values) of edges from one node to all nodes within its corresponding network divided by the number of all edges in the network (for n nodes, there are n*[n − 1]/2 possible edges in a fully connected network)
- Inter‐network connectivity estimate comprising the sum of edge weights from one node to all nodes outside its corresponding network divided by the number of the respective edges.

Thus, for each node, six different strength values were calculated, three intra‐network (SC, FC_pos_, and FC_neg_) and three inter‐network estimates (SC, FC_pos_, and FC_neg_), in total comprising 2400 connectivity values (400 nodes × 6 strength values) for each subject. Of note, density values for functional inter‐ and intra‐network parameters can be found in Table S2.

### 2.4 Cognitive performance

All subjects underwent comprehensive neuropsychological assessment addressing a wide range of cognitive functions including the domains of attention, episodic and working memory, executive as well as language functions (for a detailed description of neuropsychological tests, see, Caspers et al., 2014; Jockwitz et al., 2017; Stumme et al., 2020). In cases of one (*n* = 31) or two (*n* = 6) missing values in the neuropsychological assessment (≥3 missing values led to exclusion, see above), they were replaced by the appropriate median (calculated separately for sex and age decades: 55–64 years, 65–74 years, 75–80, and >85 years). Principal component analysis (PCA) was applied to reduce the neuropsychological data to one cognitive performance component (COG). Previously, data was tested on suitability for PCA, using the Kaiser–Meyer–Olkin (KMO) index indicating suitability of data for PCA (KMO = 0.89; Tabachnick et al., 2007).

### 2.5 Statistics

To unveil FC and SC patterns that are, together with cognitive performance, characteristic for the older adults' aging process, we performed a partial least square regression (PLSR; Mevik et al., 2018) with COG, whole‐brain region‐wise SC, FC_pos_, and FC_neg_ values as predictor variables (corrected for sex and education) and chronological age as the response variable. PLSR is a multivariate statistical approach that has the advantage of effectively dealing with multiple predictor variables that may even extend the number of observations and depict high collinearity (Haenlein & Kaplan, 2004; Krishnan et al., 2011; McIntosh & Lobaugh, 2004). In PLSR, predictor variables are decomposed into a smaller set of independent components (using a nonlinear iterative partial least squares algorithm, NIPALS) on which a least square regression is performed to define components that are maximally correlated with the response variable. Hence, within one component predictor variables (COG, SC, FC_pos_, and FC_neg_) are clustered in a unique combination, such that a unique amount of variance is used to explain the highest possible amount of variance in age. In the following, the components are called “aging profiles,” that is, comprising both the connectivity predictors (connectivity profile) and the cognitive performance predictor.

To extract the number of components that explain a significant proportion of variance in age without overfitting the model, a permutation approach with cross‐validation is included in PLSR (Mevik et al., 2018; Mevik & Wehrens, 2015). Thereby, PLSR is repeatedly calculated with the inclusion of different numbers of components, each run omitting one individual and determining cross‐validated residual values (leave one out cross‐validation to depict the difference between the actual response and predicted response value). For each model (different number of components included), the root mean squared error of prediction (RMSEP) is calculated by summing all squared prediction errors. Based on the derived RMSEP for each component, a permutation test is used to determine the number of components to be included until there is no further significant improvement in predictive performance [α = .01; for a detailed description of PLSR, also see, Mevik and Wehrens (2015) and Mevik et al. (2018)].

For each component, PLSR provides loading values for each predictor variable indicating the association between predictor and age (whether the predictor shows age‐related connectivity increases or decreases). Components‐derived loading values then reveal how region‐wise connectivity estimates are combined, that is, how they are together age‐characteristic. Furthermore, with COG being included as a predictor variable, components‐derived connectivity profiles can additionally be related to cognitive performance.

To assure that results of the PLSR are applicable and robust across multiple datasets, we split the whole sample into 1000 different training (80%, *n* = 458) and test datasets (20%, *n* = 115), performed PLSR on the training datasets and applied the derived model to the remaining test datasets to predict age (based on their predictor variables). Further, to validate that PLSR on real data performs significantly better as compared to random data, we reran all analyses with 1000 null models (created by randomly scrambling age and connectivity estimates) and compared model performances. All PLSRs were additionally performed with only the inclusion of cognition and FC or cognition and SC. To ensure that the results are not dependent on the specific sample split that was used, we also performed the same analyses by using three other sample divisions (90/10%, 70/30%, and 60/40%). Finally, to ensure that results were robust to participant's health status, we reran PLSR with the additional inclusion of available covariates indicative of our participants' state of health [total grey matter volume (ml), white matter lesions (mm^3^), blood pressure (mmHg), blood glucose concentration (%), and BMI (body mass index)]. For details, see Figure S1 and Table S3.

After model validation, the different PLSR‐derived components, that is, aging profiles, were inspected. As stated above, for each of the 1000 permutations, PLSR provides loading values for each predictor variable in each component. To make the strength of loadings more easily interpretable across networks, we calculated network‐wise average mean loading values (the average across all mean loading values within one network). With regards to previous literature indicating that the frontal lobe is structurally more sensitive to age‐related decreases as compared to the rest of the brain, we statistically tested this by calculating the average mean loading values of regions located within the frontal lobe and compared these to the average mean loading values located in the rest of the brain using an undirected two‐sample *t*‐test (Figure S6).

## 3 RESULTS

### 3.1 Cognitive performance

**FIGURE 1:**
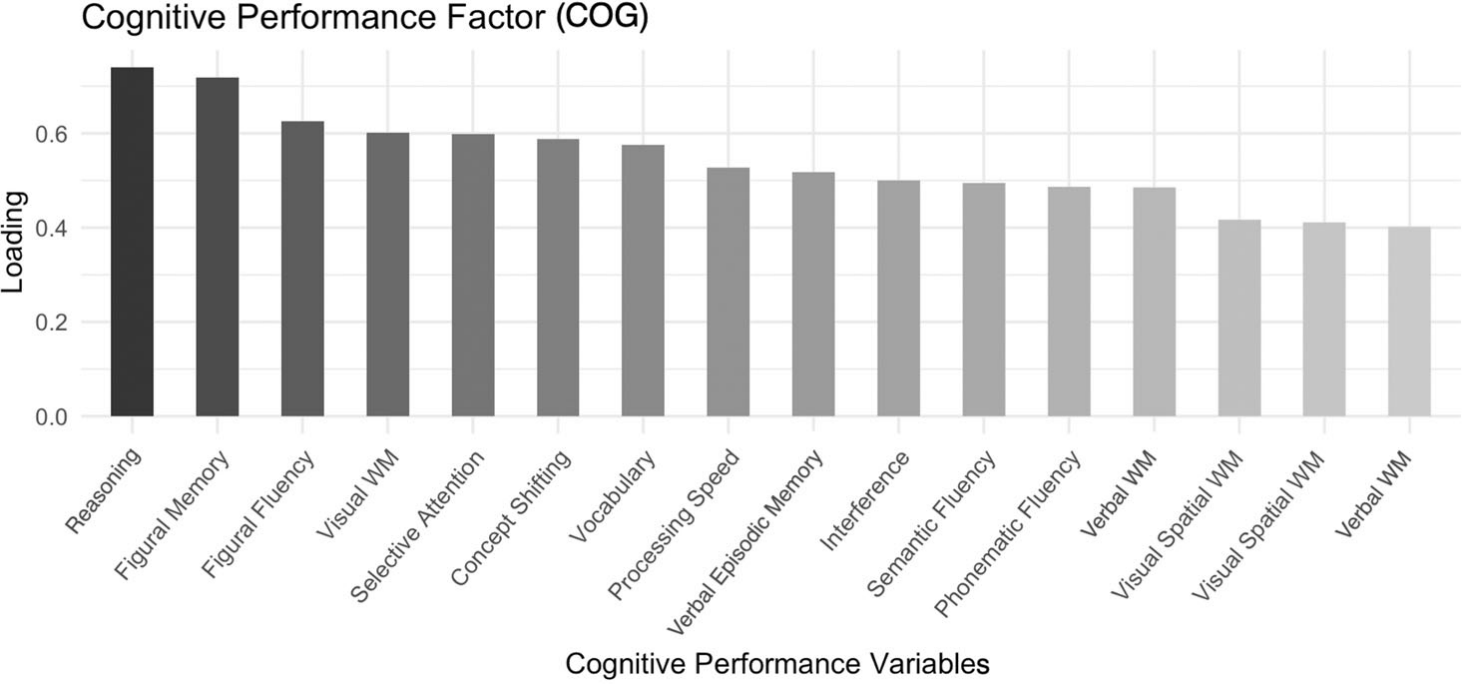
PCA derived factor loadings for the cognitive performance, ordered descendent according to the strength of loading. STM, short‐term memory; WM, working memory

Using PCA, we reduced the cognitive performances across 16 different cognitive test scores into one comprehensive cognitive performance component (Figure 1). Relating the cognitive factor to age, sex, and education revealed a significant negative correlation with age (*r* = −.44, *p* < .001, corrected for sex and education), a significant positive relation to education (*r* = .40, *p* < .001, corrected for age and sex), and no sex‐related performance differences (*F* = 2.31, *p* = .129, corrected for age and education).

### 3.2 PLSR—Model validation

**FIGURE 2:**
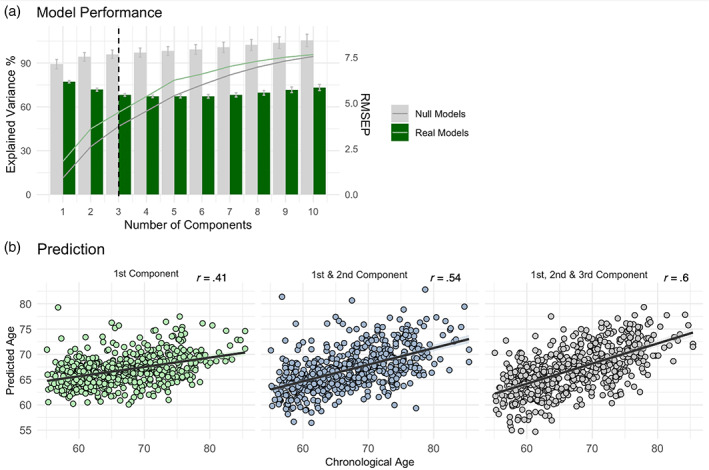
PLSR model description. (a) Model performance across 1000 real models (green) or null models (grey): RMSEP (SD) as bars and explained variance in age (%, *R* ^2^) as lines including up to 10 components. Dashed line indicating the utilized model in the current study. (b) Prediction accuracies derived from applying the PLSR model on 80% of the sample to unseen test datasets (20% of the sample): Correlation between predicted and chronological age including the information of only the first component, the first and second component, or all three components. Individual score values depict the mean scores across 1000 permutations

Results from the PLSR model validation revealed that the inclusion of three components appears optimal in the current context, that is, the model explains sufficient variance, while preventing an overfitting of the model. Importantly, PLSR on real models performed significantly better as compared to null models [RMSEP_real(SD)_ = 5.45 (.07); RMSEP_null(SD)_ = 7.68 (.23); Figure 2a, Table 2]. Additively including information of the first, second, and third components revealed a successive increase of explained variance in age (first: *R* ^2^ = 22.7%; second: *R* ^2^ = 44.9%; third: *R* ^2^ = 56.2%) and an increasing correlation between predicted and chronological age (first: *r* = .41, *p* < .001; second: *r* = .54, *p* < .001, third: *r* = .6, *p* < .001; Figure 2b).

**TABLE 2:** Results of PLSR based on real or null models: RMSEP, % variance used of the predictor variables (SD), the % explained variance in age (SD) by the inclusion of up to 10 components

Component	1	2	3	4	5	6	7	8	9	10
*Real models*										
RMSEP	6.18 (0.07)	5.74 (0.09)	**5.45 (0.07)**	5.37 (0.08)	5.38 (0.09)	5.38 (0.1)	5.45 (0.12)	5.57 (0.13)	5.73 (0.16)	5.86 (0.17)
% used variance COG/connectivity (SD)	8.26 (0.3)	12.9 (0.8)	**19.51 (0.92)**	22.11 (0.36)	24.04 (0.36)	27.04 (0.7)	28.73 (0.76)	29.94 (0.45)	31.07 (0.27)	31.98 (0.23)
% explained variance age (SD)	22.71 (1.18)	44.93 (2.47)	56.18 (1.09)	67.05 (1.6)	78.24 (0.96)	82.46 (1.52)	87.68 (0.65)	91.24 (0.9)	93.96 (0.52)	95.78 (0.35)
*Null models*										
RMSEP	7.14 (0.25)	7.54 (0.24)	7.68 (0.23)	7.77 (0.25)	7.85 (0.24)	7.94 (0.27)	8.06 (0.28)	8.19 (0.3)	8.31 (0.32)	8.44 (0.34)
% used variance COG/connectivity (SD)	6.67 (1.87)	21.16 (1.36)	32.41 (1.5)	41.62 (1.54)	46.15 (1.3)	51.09 (0.87)	55.77 (0.7)	58.67 (0.5)	61.01 (0.35)	63.05 (0.19)
% explained variance age (SD)	11.34 (5.09)	32.74 (5.38)	46.63 (3.87)	57.03 (3.86)	67.62 (2.63)	75.06 (2.26)	81.8 (1.91)	87.18 (1.32)	91.38 (0.79)	94.47 (0.55)

*Note*: The cross‐validated permutation approach of PLSR (Section 2) revealed three components to explain enough variance in an age without overfitting the model.

Of note, the inclusion of various covariates addressing the participants' health status did not result in any significant alterations of the presented effects (Figure S1, Table S3). Further, performing PLSR on different training and test sample sizes (60%/40%, 70%/30%, 80%/20%, 90%/10%) revealed highly comparable results across all sample splits (Figure S2, Tables S4–S6). Finally, PLSR based on either cognition with SC or cognition with FC revealed both models to significantly outperform null models, though with better model performances based on SC as compared to FC (Figure S2, Tables S5 and S6).

### 3.3 PLSR: Aging profiles

**FIGURE 3:**
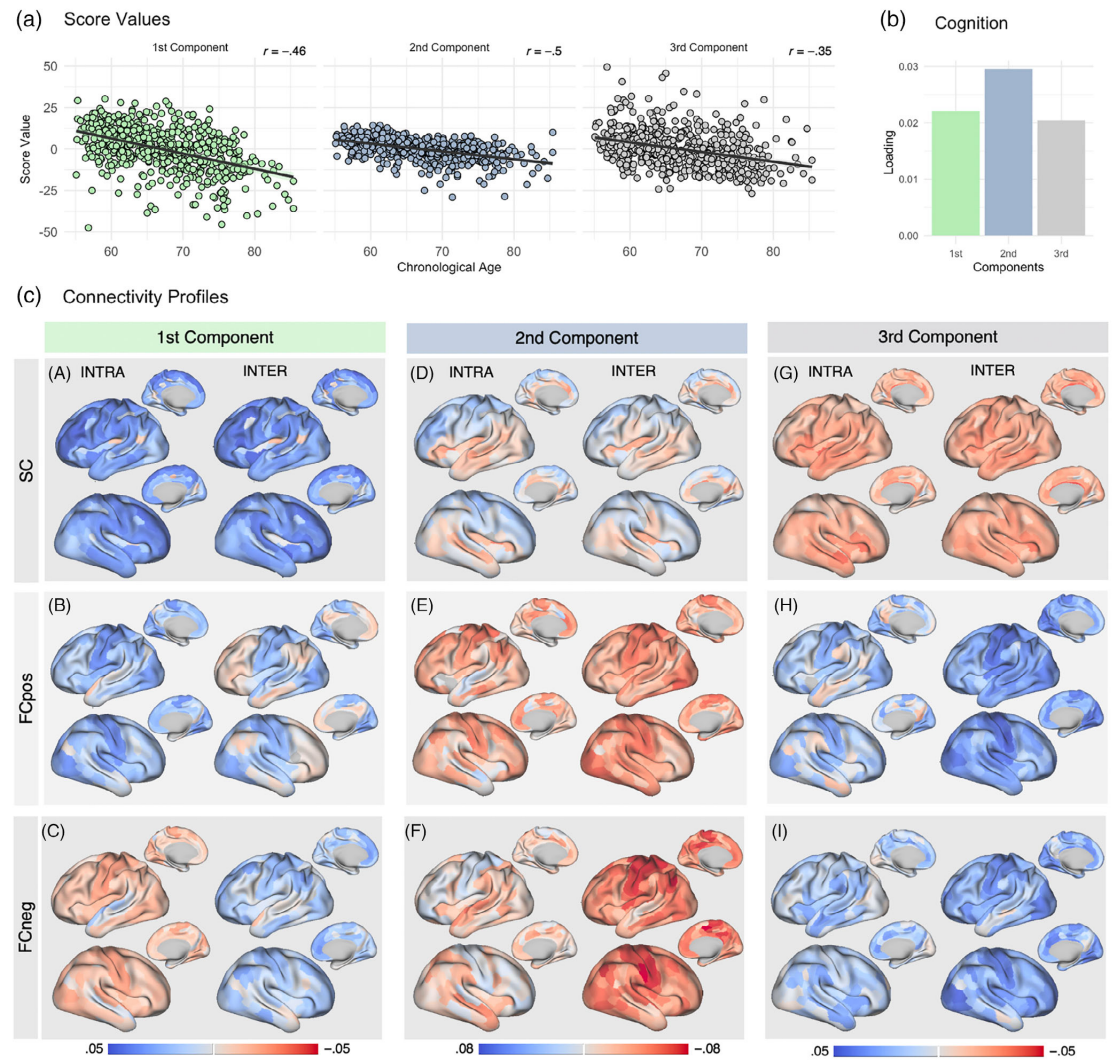
(a) Model derived individual score values for the first, second, and third components in relation to the participant's chronological age. (b) Loading values for cognitive performance in the first, second, and third components: Higher loadings indicate lower cognitive performance at higher ages. (c) Region‐specific loading values for the first (A, B, C), second (D, E, F), and third component (G, H, I): Intra‐ and inter‐network SC (A, D, G), FC_pos_ (B, E, H), and FC_neg_ (C, F, I) plotted onto the brain surface. Blue colors indicate lower and red colors higher connectivity values being characteristic for higher ages

The PLSR model validation revealed the variance in age to be described by three different components, that is, aging profiles. Within each component, predictor variables (COG and connectivity estimates) were combined in a unique way such that they show the highest possible correlation with age. All components show a negative correlation with age (first component: *r* = .46, *p* < .001, second component: *r* = .5, *p* < .001, third component: *r* = .35, *p* < .001; Figure 3a). Within each component, this age‐related shift can comprise age‐related increases or decreases of predictors, determined for each predictor variable separately and indicated by the respective loading value.

We found cognitive performance to be depicted by positive loading values in all components with an emphasis on the second component (first component: COG_mean(SD)_ = .022 (.002), second component: COG_mean(SD)_ = .030 (.005), third component: COG_mean(SD)_ = .021 (.002); Figure 3b) indicating that higher ages are related to lower global cognitive performance, especially in the second component.

**FIGURE 4:**
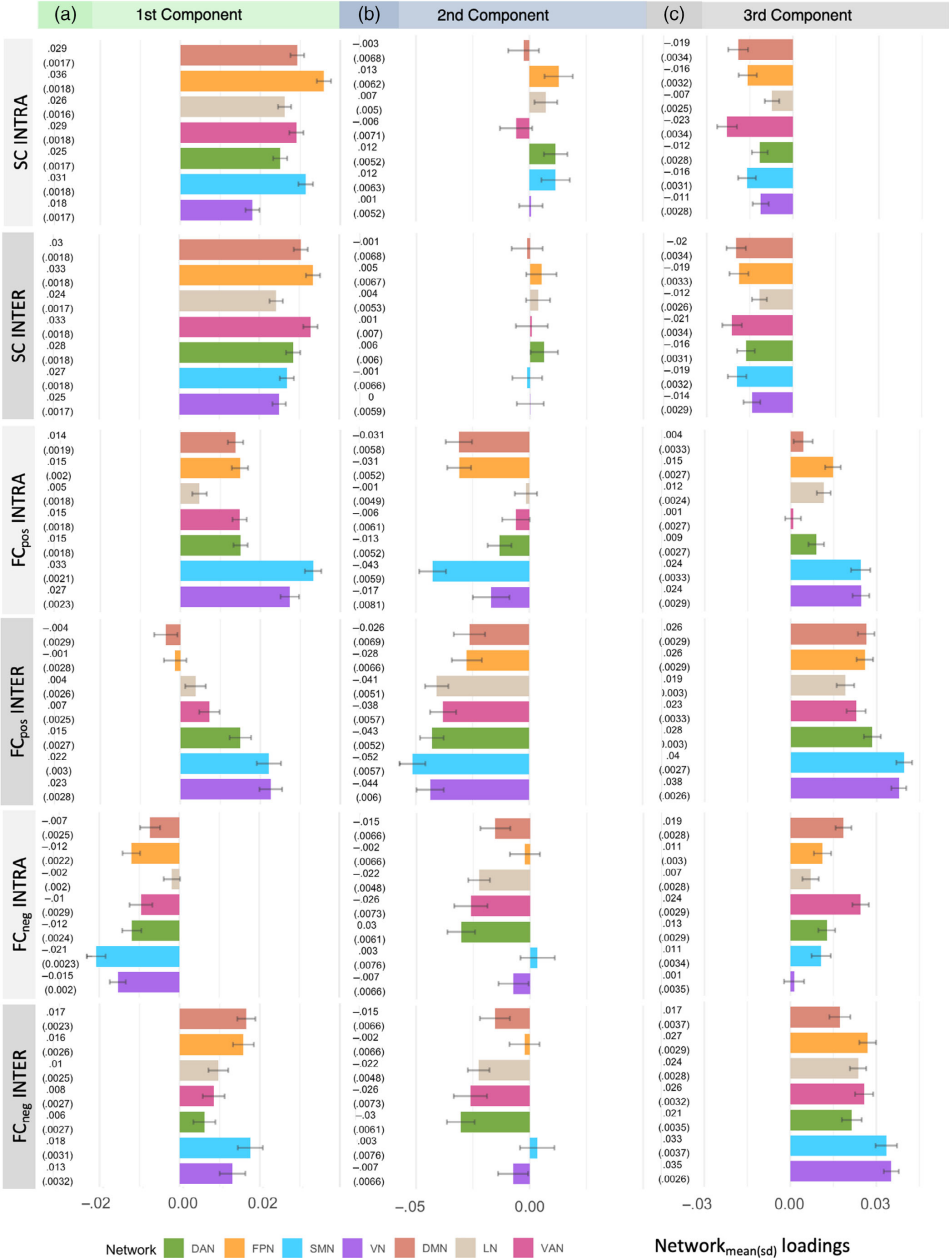
Network‐wise mean loading values (SD) for the first, second, and third components visualized as bar plots: Inter‐ and inter‐network SC, FC_pos_, and FC_neg_ (colored according to their respective network, from top to bottom: Brown = DMN, orange = FPN, grey = LN, pink = VAN, green = DAN, blue = SMN, violet = VN)

Regarding the connectivity profiles, that is, how region‐wise connectivity predictors are combined in each aging profile, we plotted region‐wise mean loading values (the mean of a predictor's loading values derived from 1000 permutations) for intra‐ and inter‐network SC, FC_pos_, and FC_neg_ onto the brain surface (Figure 3c). While positive loading values indicate age‐related connectivity decreases (blue color), negative loading values show the opposite association, that is, age‐related connectivity increases (red color). Overall higher loading values indicate a stronger association with age underpinning these connectivity estimates to be highly age‐characteristic. In the following sections, the three derived age‐related connectivity profiles will be successively described by referring to the concurrent effects of SC, FC_pos_, and FC_neg_. As outlined in Section 2, network‐wise mean loading values were calculated to make the strength of loadings more easily interpretable across networks (Figure 4). For results on region‐wise loading, which are informative about the distribution of loadings within networks, refer to Figures S3–S5.

### 3.4 First component

In the first component, 8% of the variance in the predictor variables is used to explain the highest variance in age (23%) indicating that this connectivity profile is most applicable to older adults. Within this component, older age is characterized by overall low SC. Looking at the region‐wise loading values for SC (Figure 3c‐A), age‐related decreases seem to particularly affect the frontal lobe. Statistically comparing loading values in frontal brain areas to the rest of the brain indeed revealed intra‐network SC (*t* = 4.7, *p* < .001) as well as inter‐network SC (*t* = 5.3, *p* < .001) to be significantly higher in frontal brain parts (Figure S6). Network‐wise, the FPN, DMN, and SMN are depicted by the strongest age‐related decreases, while the FPN, DMN, and VAN are most sensitive in terms of age‐related inter‐network SC decreases. Because the FPN, SMN, DMN, and VAN have regions located in both frontal as well as more posterior brain parts, SC decreases seem to affect regions in the frontal lobe independent of their network affiliation.

This age‐related decrease in SC is accompanied by overall age‐related decreases of the intra‐network FC_pos_ (Figure 3c‐B) and increases of intra‐network FC_neg_ (Figure 3c‐C) pertaining to all networks distributed across the whole brain. Hence, while coactivations of regions within networks decrease with higher age, their anticorrelations show an opposite trajectory. Thereby, loading values of primary processing networks are notably high [FC_pos_: VN_mean(SD)_ = .027 (.002), SMN_mean(SD)_ = .033 (.002); FC_neg_: VN_mean(SD)_ = .021 (.002), SMN_mean(SD)_ = .015 (.002), Figure 4a] indicating the strongest age‐related differences in both positive connections and anticorrelations. Concurrently, the inter‐network FC_pos_ shows age‐related decreases (Figure 3c‐B). This, however, is not applicable to higher order networks: regions inside the VAN, LN, FPN, and DMN show age‐related increases, mainly pertaining to the FPN and DMN [FPN_mean(SD)_ = −.001 (.003), DMN_mean(SD)_ = −.004 (.003); Figures 3c‐B and 4a]. Anticorrelations show decreases across all networks indicating less network‐specific coactivations, but more simultaneous activations of regions from different networks.

Cohesively, the first connectivity profile implies age‐related decreases in SC and FC_pos_ accompanied by increasing anticorrelations within all networks and across the whole brain. Specifically, as the age‐related decline of SC affects the whole brain, we see age‐related decreases of the intra‐network FC_pos_ in particularly primary processing networks together with age‐related increases of inter‐network FC_pos_ of higher‐order networks. Regarding cognition, increasing age is associated with decreasing performance that is comparable to the third component and slightly less advanced compared to the second component [first component: COG_mean(SD)_ = .022 (.002), second component: COG_mean(SD)_ = .030 (.005), third component: COG_mean(SD)_ = .021 (.002); Figure 3b].

### 3.5 Second component

In the second component, another 4% of the variance in the predictor variables is clustered such that it explains another 22% of the variance in age. In contrast to the first component, the second component comprises a connectivity profile in which age‐related SC decreases only affect the frontal lobe and parts of the parietal lobe, while regions within the temporal, and occipital lobe and the insula remain rather stable (Figure 3c‐D). This is applicable to both, intra‐ and inter‐network SC. Accordingly, SC decreases in frontal brain areas are again significantly stronger as compared to the rest of the brain (intra‐network SC: *t* = 2.5, *p* = .015; inter‐network SC: *t* = 2.7, *p* = .008, Figure S6). Inspecting the loading values across networks (Figure 4b, Figure S4), each network comprises regions with positive as well as negative loading values indicating age‐related differences of regions to be rather independent of their network affiliation.

In terms of FC, the second component FC_pos_ is (in contrast to the first component) overall high in older adults (Figure 3c‐E). This is applicable to the FC_pos_ within‐ as well as between‐networks, with an emphasis on the SMN (intra‐network: SMN_mean(SD)_ = .043 (.006); inter‐network: SMN_mean(SD)_ = .052 (.006); Figure 4b). Furthermore, especially between networks, anticorrelations show age‐related increases, indicating that networks do not only show more coactivations, but also higher anticorrelations in higher ages (Figure 3c‐F). Of note, comparing the intra‐network SC and FC (left column in Figure 3c‐D, E, F) one can see that regions which remain rather stable in SC across age (superior temporal lobe and insular) seem to show comparably low increases in FC_pos_ and FC_neg_. In contrast, regions with stronger age‐related decreases in SC show stronger increases in both, FC_pos_ as well as FC_neg_. Remarkably, the second component is associated with the strongest age‐related differences in cognitive performance as indicated by the highest COG loading value (Figure 3b).

### 3.6 Third component

As compared to the first and second components, the third component explains less variance in age (11%) by using 7% of the variance of the predictors. Therefore, this connectivity profile is comparably less representative for older adults. Here, the SC show overall negative loading values indicating a stable SC system across age (Figure 3c‐G) with no age‐related SC decreases affecting either the intra‐ or inter‐network SC of any networks (Figure 4c). Remarkably, this overall stable SC profile is clustered together with overall low FC_pos_ (Figure 3c‐H) as well as low FC_neg_ (Figure 3c‐I). Thereby, the intra‐ and inter‐network FC_pos_, as well as inter‐network FC_neg_ of primary processing networks, show the strongest relations to age, indicating that these networks are most age‐characteristic and showing the strongest age‐related decreases [intra‐network FC_pos_: VN_mean(SD)_ = .024 (.003), SMN_mean(SD)_ = .024 (.003); inter‐network FC_pos_: VN_mean(SD)_ = .038 (.003), SMN_mean(SD)_ = .040 (.003); inter‐network FC_neg_: VN_mean(SD)_ = .035 (.003), SMN_mean(SD)_ = .033 (.004); Figure 4c]. In terms of cognitive performance, this connectivity profile is similar to the first component accompanied by cognitive performance decreases (Figure 3b).

## 4 DISCUSSION

As we age, the functional connectome undergoes a process of reorganization that manifests itself in a shift from segregated to more integrated brain networks and which was found to be relevant in terms of cognitive performance. The causes of this functional restructuring are not yet fully understood, but are related to differences in SC. Since SC is the underlying scaffold for information exchange between regions, age‐related SC differences may explain age‐related FC reorganizations. Here, we took advantage of a large cohort of older adults and performed a multivariate statistical approach (PLSR) on the participant's regions‐wise FC, SC and global cognitive performance. Specifically, we examined how region‐wise FC and SC are together age‐characteristic and related to cognitive performance. In doing so, we aim to contribute to the understanding of age‐related functional restructuring by considering SC differences that are associated with it.

Results of PLSR indicate that the variance in age is explained by three different aging profiles. Of note, sensitivity analyses indicate these aging profiles to be robust across multiple sample splits and independent of the overall health status of participants. In line with previous research PLSR with only cognition and SC explained more variance in age as compared to cognition and FC (Cole, 2020). Inspecting the aging profiles in detail revealed interesting interrelations of region‐wise FC and SC estimates, which will be discussed as follows.

With regards to previous research, we assumed age‐related decreases in SC across the whole brain with a particular focus on the frontal lobe. This is exactly what is captured by the first aging profile (first component). Here, SC across the whole brain was characteristic for higher ages and the strongest age‐related decreases in SC pertain to the frontal lobe. These effects were very similar not only for the two hemispheres, but also for SC within and between networks. Hence, decreases affect the SC between any regions (in all networks), but especially those located in the frontal lobe. Previous results on lifespan changes indicate that white matter of the frontal lobe is particularly vulnerable to the aging process showing the greatest deteriorations across ages while white matter of temporal and occipital regions seem to be relatively preserved (Antonenko & Floel, 2014; Gunning‐Dixon et al., 2009; Rojkova et al., 2016; Salat, 2011; Salat et al., 2005; Zhao et al., 2015). In the current sample of older adults, we found the first component to capture SC decreases that affect the whole brain. Thereby, decreases in frontal brain areas are indeed most age‐specific, but the rest of the brain is additionally affected, though to a somewhat lesser extent. These effects may represent a more advanced picture of aging (in older adults as compared to lifespan samples) that has additionally affected SC in parietal and temporal regions.

This SC profile is clustered together with a FC profile that very much matches the typical FC aging pattern described in previous research on older adults (Edde et al., 2021; Perry et al., 2017; Stumme et al., 2020; Zonneveld et al., 2019). The functional profile of the first component is in line with our hypothesis that higher age is characterized by lower intra‐network FC of particularly primary processing networks together with a higher integration between higher‐order networks. The strongest age‐related decreases of intra‐ and inter‐network FC pertain to the VN and SMN indicating that in older adults a reduced FC_pos_ of particularly primary processing networks is characteristic for higher ages. Concurrently, higher order networks (especially the DMN and FPN) show higher positive inter‐network FC at higher ages, perfectly reflecting the assumed age‐related shift towards a stronger network integration of higher order networks (Betzel et al., 2014; Edde et al., 2021; Ferreira et al., 2016; He et al., 2020; Stumme et al., 2020; Tsvetanov et al., 2016; Varangis et al., 2019). Complementary to positive FC, FC anticorrelations within networks show overall age‐related increases in FC which could indicate that regions within a network work less coherently at higher ages. However, these results must be viewed with caution, as anticorrelation within networks are rather unlikely and may be caused by a topographical deviation of older adults to the younger adults parcellation used in the current study (further discussed in the methodological considerations). In turn, anticorrelations between networks decrease, indicating a reduced ability to deactivate brain networks while activating another, and thus leading to a shift towards greater inter‐network integration (Edde et al., 2021; Ferreira et al., 2016; Keller et al., 2015; Spreng et al., 2016). Current research agrees that lower intra‐network FC is associated with lower performances, meaning that less coherent networks result in poorer cognitive functioning (Ewers et al., 2021; Fjell et al., 2015; Marques et al., 2016; Stumme et al., 2020).

In turn, inter‐network FC increases can be interpreted in two ways: as a compensatory attempt or a dedifferentiation process. In terms of compensation, the additional functional recruitment of higher order networks may be understood as the attempt to more intensively involve additional control processes (e.g., monitoring, introspection, and attention processes) to maintain cognitive performances despite a decay of network coherence. A higher recruitment of brain regions may be accompanied by increasing wiring costs, but may also be accompanied by a higher cognitive reserve, that is, performance maintenance (Festini et al., 2018; Franzmeier et al., 2018). As discussed in Stumme et al. (2020), specific coactivations may indeed be beneficial for cognitive maintenance. However, with an increasing number of coactivations, specific access to the auxiliary functions and thus the compensatory purpose of the system may be lost and replaced by a rather dedifferentiated system. A functionally dedifferentiated system is characterized by a reduced distinctiveness of activity patterns throughout the brain (Edde et al., 2021; Ferreira et al., 2016; Keller et al., 2015; Spreng et al., 2016) limiting the access to specific cognitive processing, which is associated with impaired performances (Monteiro et al., 2019; Spreng & Turner, 2019). The first component is accompanied by age‐related decreases in cognitive performance indicating that the additional recruitment of higher order networks during rest cannot hinder a cognitive decline. In view of the large age range (55–85 years), the strong cognitive changes in older subjects (Hedden & Gabrieli, 2004; Salthouse, 2019) and the widely affected SC decreases, a halt of cognitive loss is not to be expected. Collectively, the first component captures a connectivity profile in which both, FC and SC show their previously described typical age‐related differences in parallel. Accordingly, this aging profile explains the most variance in age.

The second component explains only slightly less variance in age as compared to the first component, indicating that there exists another aging profile that is particularly age‐characteristic in older adults. Here, the overall SC is less affected by age with only the frontal lobe showing age‐related decreases, while the parietal and occipital lobes remain stable. As discussed above, this may comprise a less advanced aging process (Antonenko & Floel, 2014; Gunning‐Dixon et al., 2009; Rojkova et al., 2016; Salat, 2011; Salat et al., 2005; Zhao et al., 2015), in which SC decreases have not yet affected the whole brain. In the case of initially decreasing SC in the frontal lobe while simultaneously large parts of the brain remain structurally intact, the brain exhibits a functionally maximally interconnected system. In fact, previous work suggests a functional over‐recruitment of brain areas to be a response to age‐related structural changes that itself would cause a poor processing of cognitive functions (Marstaller et al., 2015; Park & Reuter‐Lorenz, 2009; Pistono et al., 2021; Reuter‐Lorenz & Park, 2014). In response to decreasing white matter pathways, the aging brain must seek alternative functional routes to maintain communication between regions (Naik et al., 2017). In this regard, overall high functional interactions could be an adaptive recalibration process resulting from the initial decline of the frontal lobe to maintain cognitive performance. Unlike the first component, the second component still has a large portion of SC paths that can be used to select alternate routes so that an exchange of information is maintained. However, because the second component is associated with the strongest age‐related cognitive decline, this supports the dedifferentiation theory, in which specific access to desired functions is reduced (Edde et al., 2021; Ferreira et al., 2016; Keller et al., 2015; Spreng et al., 2016). As discussed above, in a compensation process we might expect more specific coactivations that recruit specific functions to maintain cognitive performance. As the recruitment of additional brain regions increases (either on purpose or due to necessary detours), increasing inter‐network FC may no longer be supported, but rather result in a decreased functional diversity of brain networks. Hence, although a compensation process may have aspired, a supportive character of increasing coactivations may at some point be replaced by a decreased functional diversity of brain networks. In this context, it is highly interesting that more and more research additionally includes time into the analyses of brain function looking at functional connectivity dynamics (FCD), that is, how the FC varies across time. It has been found that with increasing age the time‐dependent variance of functional states, called metastability, declines (Battaglia et al., 2020; Lou et al., 2019; Naik et al., 2017; Xia et al., 2019). Here, the functional activity is characterized by reduced differentiated activity states, meaning that a high proportion of functional systems are activated in parallel. A lower metastability is, thereby, characterized by a lower ability of the functional system to transition between different cognitive states, that is, if the whole system is similarly activated, the potential to switch between states diminishes. This is thought to reduce the capacity to also behaviorally switch between concepts and to slow the rate of functional adaptations to external influences (Escrichs et al., 2021; Lee et al., 2019; Xia et al., 2019). Computational models showed that reduced metastability is a response to SC decline (Deco & Kringelbach, 2016; Lavanga et al., 2022; Naik et al., 2017). Hence, the functionally highly interconnected system found in the second component could point towards a low capacity to switch between functional states potentially resulting from the incipient SC decline and would explain the strongest association with cognitive performance decline. Including FCD estimates in this context, thus, would be highly promising for future research.

It remains open why the brain associated with the most severe SC decline (as in the first component) does not show a highly interconnected functional system. Participants with minor SC deterioration may experience an onset of cognitive decline, that is find the everyday tasks more difficult, but still strive to maintain cognitive performance, which may then be addressed by an increase in functional interconnectivity (Gaviria et al., 2021). However, with regards to the “Compensation‐related utilization of neural circuits hypothesis” (Reuter‐Lorenz & Cappell, 2008), the functional capacity to respond to increasing task difficulty is exhausted at some point and the attempt to compensate for increasing task complexity by functional overactivation is no longer even considered. Further, an overall reduced SC in the first component limits the possibility of alternative routes and may logistically not allow information to be relayed via many different regions.

Following the course of descending severity of SC decline from the first over the second to the third component, the third component reflects a connectivity profile which we may consider as well preserved. In this case, higher age is depicted by comparably high SC, while the overall FC is low. Overall high SC points to a well‐preserved underlying architecture that enables an efficient exchange of information between regions while consuming as little energy as possible (Lynn & Bassett, 2019). The associated resting brain exhibits rather weak FC both within and between all networks. Higher overall communication in the brain, that is, connectivity, requires higher energy consumption (Tomasi et al., 2013). At the same time, a highly interconnected functional system reduces the ability to efficiently switch between brain states (Chan et al., 2014, 2017; Colcombe et al., 2005; Goh, 2011; Nashiro et al., 2017; Park et al., 2004), which is associated with lower cognitive performance (Lavanga et al., 2022). Accordingly, the functional connectivity system of this third component could reflect a rather low‐energy state of the resting brain, which at the same time may involve a high ability to efficiently adapt to external stimuli. However, this connectivity profile is comparatively less representative in older adults, which is plausible in light of previous results showing a continuous SC decline into old age (Cox et al., 2016; Gunning‐Dixon et al., 2009; Li et al., 2020).

Collectively, we found three different connectivity profiles to be related to age in older adults. Each connectivity profile is depicted by different severity of SC decline. While a well‐preserved SC system is accompanied by a comparably low interconnected functional system, a decline in SC seems to go along with an increase in the brain FC. The functionally highest interconnected system is present when the underlying white matter pathways are only slightly damaged. This could indicate that an increasing FC is the reaction to an incipient decline of the underlying SC construct, which logistically allows the transmission of information in various detours. However, we found that the highest interconnected functional system was associated with the greatest decline in cognitive performance, indicating a shift towards higher network integration to represent a dedifferentiation process. In fact, the compensation and dedifferentiation theories do not cancel each other out. Instead, an interlocking process in which a beneficial compensation process is replaced by a steadily decreasing diversity of functional systems may be conceivable.

### 4.1 Methodological considerations

The results of the present study are based on a cross‐sectional design. The current cross‐sectional design has the advantage of a large sample size representative of and thus, largely generalizable for, the general older population in West Germany. For capturing the intraindividual age‐related changes in the relationship between SC and FC, however, longitudinal studies are warranted.

A potential limitation of the current study pertains to the specific functional network parcellation used, which is based on resting‐state data from younger adults. Methods for such imaging‐based brain parcellations improved considerably over the recent decade (Eickhoff et al., 2018). Nevertheless, so far, no whole brain network parcellation based on older adults exists integrating both, structural and functional information. Within the current study, we chose the current parcellation based on previous work on functional (Schaefer et al., 2018) and anatomical data (Varikuti et al., 2018) indicating fine‐grained parcellations of 300–600 nodes to be optimal. Especially using fine‐grained parcellations, however, transformation procedures between image modalities could influence inter‐subjects' variance. Hence, changes in the parcellation granularity and further, the inclusion of subcortical structures would be interesting for future studies focusing on SC–FC relations during aging and their link to cognitive performance.

SC evolves, rearranges, and strengthens in developmental stages, after brain injuries as well as across the lifespan as a result of, for example, learning processes (Fields, 2005; Salat, 2011; Yeatman et al., 2014). However, in older ages, increases in SC are rather unlikely and may point to yet unresolved methodological constraints. In addition, tractography on diffusion imaging data is not a direct measurement, but only an estimation of anatomical connectivity (Sotiropoulos & Zalesky, 2019) known to under‐represent long‐distance white matter connections (de Reus & van den Heuvel, 2013). Across the aging process the paucity of long‐distance connections even increases, which may foster increasing short‐range connections (Puxeddu et al., 2020; Zhao et al., 2015). So far, a ground truth for structural connectomes has not yet been developed. To optimally picture the biological SC, we conducted streamlined filtering as an additional step in diffusion MRI denoising (Smith et al., 2015). Furthermore, particularly for SC, network properties are known to depend on the methodology applied, which potentially makes specific network results less generalizable (Qi et al., 2015).

As compared to previous studies on age prediction our model explains less variance in age. Although the validation process revealed our PLSR model to perform significantly better as compared to random data underpinning the model's prediction ability. So far, the optimal method for age prediction is still under debate (Smith et al., 2019). Predictions were found to perform best using structural brain volume data (Cole et al., 2017; Cole & Franke, 2017; Franke et al., 2010; Liem et al., 2017), while age prediction on connectivity data was found to perform significantly lower explaining about 40%–60% of the variance in age (Dosenbach et al., 2010; Han et al., 2014; Li et al., 2018; Vergun et al., 2013). With respect to the current study, the intended restriction to region‐wise connectivity estimates limits the informative value for age prediction to only particular connectivity values. The inclusion of more specific connectivity measures, for example, individual edge weights, may potentially increase prediction accuracy.

Finally, it should be noted that FC anticorrelations imply a qualitatively distinct type of interaction between brain regions, which is not yet clearly interpretable (Chai et al., 2012; Fornito et al., 2013; Murphy & Fox, 2017). Negative correlations may be artificially induced, when using global signal regression in functional imaging preprocessing (Fox et al., 2009; Murphy et al., 2009; Murphy & Fox, 2017). Therefore, results on negative correlations have been included in this study as additional complementary evidence for the general relation between FC and SC, without demanding clear interpretability on its own.

## 5 CONCLUSION

The normal aging process is accompanied by a restructuring of the functional connectome, characterized by a shift from more segregated to more integrated brain networks which was found to be important for changes in our cognitive performance. Causes of the functional restructuring remain unclear, but may be associated with age‐related SC differences, depicting the underlying scaffold for information exchange between regions. By performing PLSR with FC and SC estimates as well as cognitive performance data from a large cohort of older adults, we investigated the interdependency of region‐wise SC and FC differences and how these are, together with cognitive performance, characteristic of older adults' age. Our results revealed three different aging profiles to be prevalent in older adults. Overall, it appears that the frontal lobe of older adults is particularly affected by aging with respect to SC showing the greatest age‐related decline. In terms of brain function, primary processing networks are most indicative of the older adult's age. In this context, the functional activity pattern seems to behave differently depending on the severity of SC deterioration. In a well‐preserved structural connectome, the brain exhibits a less interconnected system at rest, characterized by particularly low connections between networks. In turn, when SC shows minor age‐related deteriorations affecting the frontal lobe, the brain exhibits a functionally maximally connected system. Because this connectivity pattern was associated with the most severe age‐related cognitive decline, a more interconnected functional connectivity system in older adults points to a process of dedifferentiation.

## FUNDING INFORMATION

This project was partially funded by the German National Cohort and the 1000BRAINS‐Study of the Institute of Neuroscience and Medicine, Research Centre Jülich, Germany. Furthermore, this project has received funding from the European Union's Horizon 2020 Research and Innovation Programme under Grant Agreement No. 945539 (HBP SGA3; SC).

## CONFLICT OF INTEREST

No competing interests were declared.

## Supporting information

Supporting Information

## Data Availability

Due to local regulations of data acquisition and usage, data of 1000BRAINS are available upon request from the responsible PI.
