# Supplementary material for: Interrelating differences in structural and functional connectivity in the older adult's brain
Source: Hum Brain Mapp. 2022 Aug 2;43(18):5543–61. doi: 10.1002/hbm.26030 (PMC9704795; doi:10.1002/hbm.26030)
Supplement: Supplementary file 1 — Supporting Information [file HBM-43-5543-s001.docx]

**Supplement**

**Supplementary Table 1.** Softwares used for preprocessing and analyzing structural and functional data.

|  | **Software or algorithms** | **URL** |
| --- | --- | --- |
| fMRI | FSL | [https://fsl.fmrib.ox.ac.uk/fsl](https://fsl.fmrib.ox.ac.uk/fsl/fslwiki/FSL) |
|  | DVARS | <https://github.com/asoroosh/DVARS> |
|  | CAT12 toolbox | <http://www.neuro.uni-jena.de/cat/> |
| DTI | FSL | [https://fsl.fmrib.ox.ac.uk/fsl](https://fsl.fmrib.ox.ac.uk/fsl/fslwiki/FSL) |
|  | CAT12 toolbox | <http://www.neuro.uni-jena.de/cat/> |
|  | ANTs | <https://stnava.github.io/ANTs/> |
|  | MRtrix | <https://www.mrtrix.org/> |
| Analyses | PLSR | <https://github.com/bhmevik/pls> |

**Supplementary Table 2.** Mean density values (ratio between existing to possible edges) for positive and negative functional connectivity within a network (INTRA) or between a network and the rest of the brain (INTER). VN = visual-, SMN = sensorimotor-, DAN = dorsal-attention, VAN = ventral-attention, LN = limbic-, FPN = frontoparietal-, DMN = default mode network.

|  | **Network** | **FCpos** | **FCneg** |
| --- | --- | --- | --- |
| **INTRA** | VN | 0.80(0.11) | 0.19(0.11) |
|  | SMN | 0.82(0.11) | 0.17(0.11) |
|  | DAN | 0.72(0.09) | 0.26(0.08) |
|  | VAN | 0.77(0.09) | 0.22(0.09) |
|  | LN | 0.66(0.09) | 0.32(0.09) |
|  | FPN | 0.69(0.08) | 0.29(0.08) |
|  | DMN | 0.66(0.06) | 0.33(0.06) |
| **INTER** | VN | 0.23(0.02) | 0.27(0.02) |
|  | SMN | 0.23(0.01) | 0.26(0.01) |
|  | DAN | 0.24(0.01) | 0.25(0.01) |
|  | VAN | 0.24(0.01) | 0.25(0.01) |
|  | LN | 0.23(0.02) | 0.23(0.02) |
|  | FPN | 0.21(0.02) | 0.28(0.02) |
|  | DMN | 0.20(0.02) | 0.29(0.02) |


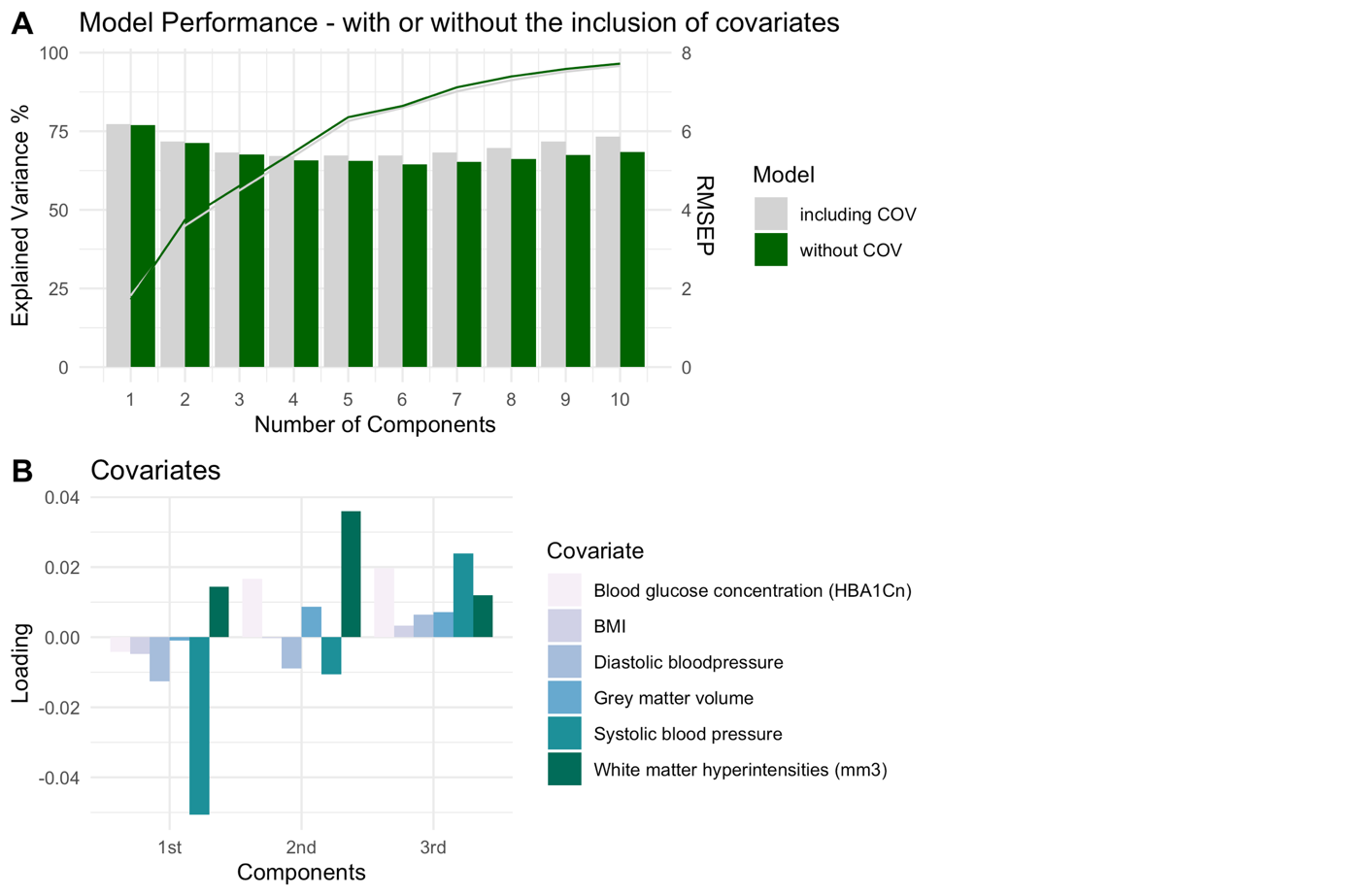


**Supplementary Figure 1.** A) Model performance based on an 80/20 split, with (grey) and without (green) the additional inclusion of covariates (COV) indicating the participants health status: RMSEP and explained variance in age (%) including up to ten components. B) Loading values of covariates in the first, second and third component. Covariates include: the participants body mass index (BMI), the brain’s total grey matter volume in ml, the total volume of white matter hyperintensities in mm3 (for methods description see below), an index of the blood glucose concentration (HbA1c) in % as well as systolic and diastolic blood pressure in mmHg. Although covariates show a relation to the participants age, including the covariates into the analyses had no effects on the estimated aging profiles.

Total white matter hyperintensity volume (WMH) was calculated using the Brain Intensity AbNormality Classification Algorithm [BIANCA (Griffanti et al., 2016)]. Before applying the BIANCA segmentation, image preprocessing was performed using FSL (<http://fsl.fmrib.ox.ac.uk/fsl>) including brain extraction, co-registration of the modalities (T1 to FLAIR), and normalization to the MNI152 standard template using linear rigid-body registration. First, a test dataset (n = 120) was generated on which a trained operator manually segmented WMH (value of 1) from non-WMH (value of 0) on the preprocessed FLAIR images. The manually segmented masks were subsequently used to train BIANCA and to evaluate its performance by comparing BIANCA estimations to the manual masks in leave-one-out tests (Griffanti et al., 2016). The metrics applied to evaluate the degree of overlap between the BIANCA estimations and the manual mask included dice similarity index (SI), voxel-level false discovery rate (FDR), voxel-level false negative ratio (FNR), cluster-level FDR, cluster-level FNR, mean total area (MTA), detection error rate (DER), outline error rate (OER) (<https://fsl.fmrib.ox.ac.uk/fsl/fslwiki/BIANCA/Userguide#Performance_evaluation>). As suggested in the current literature, we selected the dice similarity index as the overlap measure with the highest importance for the decision regarding the option selection. Further, a low FNR was prioritized to achieve a high sensitivity of lesion detection. The optimized configurations used for the calculations of WMH in the current study were: FLAIR+T1 (FLAIR as base modality), threshold = 0.9, spatialweight = 1, no patch, no border option, different number of training points for WMH (5000) and non-WMH (25000). The trained algorithm was then applied to all subjects to extract subject-wise WML volumes.

**Supplementary Table 3.** Model performance based on an 80/20 split with the additional inclusion of covariates indicating the participants health status: RMSEP and explained variance in age (%) and used variance in connectivity (%) including up to ten components. For visualization see Supplementary Figure 1.

| Component | **1** | **2** | **3** | **4** | **5** | **6** | **7** | **8** | **9** | **10** |
| --- | --- | --- | --- | --- | --- | --- | --- | --- | --- | --- |
| RMSEP | 6,16 | 5,7 | 5,41 | 5,26 | 5,25 | 5,16 | 5,22 | 5,29 | 5,4 | 5,48 |
| % expl. var. age | 9,1 | 12,93 | 20,30 | 23,08 | 24,59 | 28,16 | 29,99 | 31,07 | 32,35 | 33,48 |
| % used var. connectivity | 21,48 | 46,82 | 57,62 | 68,35 | 79,47 | 83,05 | 88,97 | 92,41 | 94,81 | 96,5 |

**
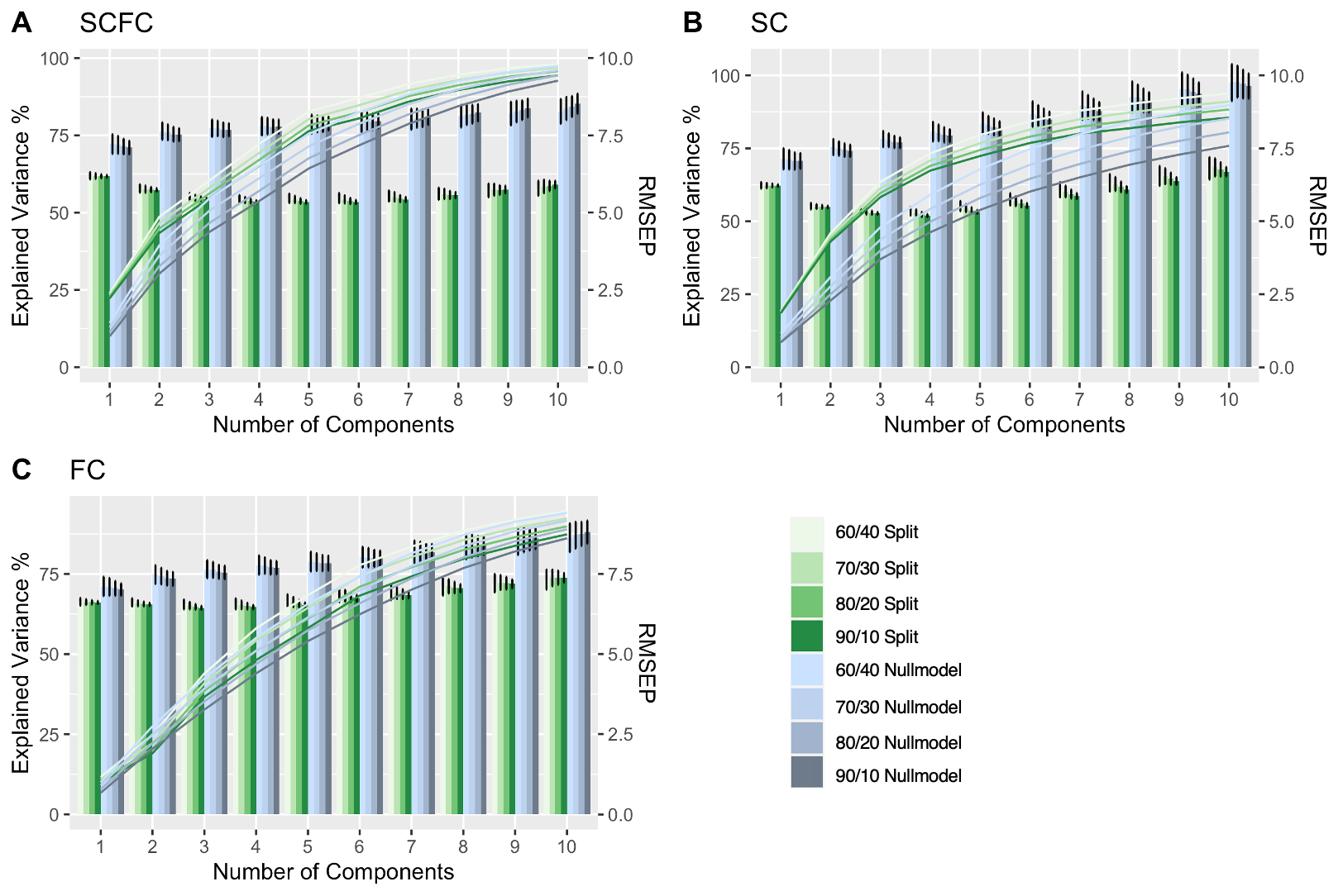
**

**Supplementary Figure 2.** Model performance across four different sample splits (60/40, 70/30, 80/20 or 90/10) based on real models (green) or null models (blue). Models included region-specific connectivity values of cognition, structure and function (A), only cognition and structure (B) or only cognition and function (C): RMSEP and % explained variance in age including up to ten components.

| SCFC | | Component | 1 | **2** | 3 | 4 | 5 | 6 | 7 | 8 | 9 | 10 |
| --- | --- | --- | --- | --- | --- | --- | --- | --- | --- | --- | --- | --- |
| 60 | Real  Model | RMSEP | 6,23 (0,11) | 5,52 (0,11) | 5,36 (0,12) | 5,34 (0,14) | 5,52 (0,18) | 5,75 (0,21) | 6,08 (0,26) | 6,32 (0,3) | 6,55 (0,34) | 6,81 (0,38) |
|  |  | % used var. connectivity | 19,18(0,97) | 23,19 (0,37) | 25,13 (0,18) | 26,97 (0,16) | 28,4( 0,19) | 29,78 (0,16) | 30,78 (0,17) | 32,1 (0,27) | 33,34 (0,25) | 34,38 (0,23) |
|  |  | % expl. var. age | 19,28(1,78) | 45,91 (1,87) | 63,52 (1,7) | 73,25 (1,38) | 79,57 (1,33) | 84,22 (1,1) | 87,88 (0,99) | 90,19 (0,92) | 92,13 (0,78) | 93,76 (0,68) |
|  | Null  Model | RMSEP | 7,22 (0,32) | 7,64  (0,28) | 7,73  (0,28) | 7,8  (0,3) | 7,86  (0,31) | 7,93  (0,32) | 8,02  (0,34) | 8,12  (0,37) | 8,21  (0,38) | 8,27  (0,4) |
|  |  | % used var. connectivity | 6,68  (1,92) | 21,04  (1,38) | 32,15  (1,53) | 41,6  (1,53) | 46,54  (1,28) | 51,5  (0,89) | 56,13  (0,65) | 59,3  (0,49) | 61,78  (0,34) | 63,86  (0,23) |
|  |  | % expl. var. age | 14,34(6,1) | 39,47  (5,99) | 54,58  (4,02) | 65,18  (3,72) | 75,48  (2,56) | 82,48  (2,18) | 88,3  (1,57) | 92,56  (0,99) | 95,52  (0,56) | 97,47  (0,32) |
| 70 | Real  Model | RMSEP | 6,18  (0,09) | 5,76  (0,11) | 5,48  (0,09) | 5,41  (0,1) | 5,43  (0,12) | 5,43  (0,13) | 5,51  (0,15) | 5,61  (0,17) | 5,74  (0,2) | 5,85  (0,21) |
|  |  | % used var. connectivity | 8,28  (0,41) | 12,79  (1) | 19,51  (1,14) | 22,17  (0,54) | 24,19  (0,44) | 27,02  (0,74) | 28,83  (0,81) | 30,12  (0,45) | 31,27  (0,31) | 32,18  (0,22) |
|  |  | % expl. var. age | 23,28(1,59) | 46,71  (3,24) | 57,98  (1,35) | 69,35  (2,08) | 80,29  (1,15) | 84,72  (1,64) | 89,57  (0,74) | 92,89  (0,87) | 95,38  (0,46) | 96,98  (0,31) |
|  | Null  Model | RMSEP | 7,19  (0,29) | 7,6  (0,26) | 7,73  (0,25) | 7,8  (0,27) | 7,87  (0,27) | 7,95  (0,29) | 8,05  (0,31) | 8,16  (0,33) | 8,27  (0,35) | 8,37  (0,37) |
|  |  | % used var. connectivity | 6,61  (1,94) | 21,07  (1,39) | 32,3  (1,53) | 41,68  (1,49) | 46,35  (1,32) | 51,21  (0,89) | 55,86  (0,65) | 58,95  (0,48) | 61,39  (0,32) | 63,44  (0,18) |
|  |  | % expl. var. age | 12,86  (5,86) | 36,17  (5,75) | 50,6  (3,98) | 61,12  (3,79) | 71,48  (2,66) | 78,7  (2,17) | 85,09  (1,65) | 89,9  (1,14) | 93,56  (0,69) | 96,11  (0,44) |
| **80** | **Real**  **Model** | **RMSEP** | **6,18**  **(0,07)** | **5,74**  **(0,09)** | **5,45**  **(0,07)** | **5,37**  **(0,08)** | **5,38**  **(0,09)** | **5,38**  **(0,1)** | **5,45**  **(0,12)** | **5,57**  **(0,13)** | **5,73**  **(0,16)** | **5,86**  **(0,17)** |
|  |  | **% used var. connectivity** | **8,26**  **(0,3)** | **12,9**  **(0,8)** | **19,51**  **(0,92)** | **22,11**  **(0,36)** | **24,04**  **(0,36)** | **27,04**  **(0,7)** | **28,73**  **(0,76)** | **29,94**  **(0,45)** | **31,07**  **(0,27)** | **31,98**  **(0,23)** |
|  |  | **% expl. var. age** | **22,71**  **(1,18)** | **44,93**  **(2,47)** | **56,18**  **(1,09)** | **67,05**  **(1,6)** | **78,24**  **(0,96)** | **82,46**  **(1,52)** | **87,68**  **(0,65)** | **91,24**  **(0,9)** | **93,96**  **(0,52)** | **95,78**  **(0,35)** |
|  | Null  Model | RMSEP | 7,14  (0,25) | 7,54  (0,24) | 7,68  (0,23) | 7,77  (0,25) | 7,85  (0,24) | 7,94  (0,27) | 8,06  (0,28) | 8,19  (0,3) | 8,31  (0,32) | 8,44  (0,34) |
|  |  | % used var. connectivity | 6,67  (1,87) | 21,16  (1,36) | 32,41  (1,5) | 41,62  (1,54) | 46,15  (1,3) | 51,09  (0,87) | 55,77  (0,7) | 58,67  (0,5) | 61,01  (0,35) | 63,05  (0,19) |
|  |  | % expl. var. age | 11,34  (5,09) | 32,74  (5,38) | 46,63  (3,87) | 57,03  (3,86) | 67,62  (2,63) | 75,06  (2,26) | 81,8  (1,91) | 87,18  (1,32) | 91,38  (0,79) | 94,47  (0,55) |
| 90 | Real  Model | RMSEP | 6,17  (0,05) | 5,74  (0,06) | 5,44  (0,05) | 5,35  (0,05) | 5,35  (0,06) | 5,35  (0,07) | 5,43  (0,08) | 5,56  (0,09) | 5,75  (0,12) | 5,91  (0,13) |
|  |  | % used var. connectivity | 8,25  (0,2) | 13,02  (0,52) | 19,49  (0,64) | 22,05  (0,25) | 23,87  (0,29) | 27  (0,71) | 28,62  (0,78) | 29,74  (0,4) | 30,9  (0,26) | 31,81  (0,23) |
|  |  | % expl. var. age | 22,29  (0,84) | 43,46  (1,61) | 54,68  (0,73) | 65,09  (1,17) | 76,43  (0,75) | 80,47  (1,5) | 85,91  (0,51) | 89,67  (0,88) | 92,45  (0,52) | 94,42  (0,34) |
|  | Null  Model | RMSEP | 7,11  (0,22) | 7,52  (0,21) | 7,68  (0,21) | 7,78  (0,23) | 7,88  (0,22) | 7,97  (0,24) | 8,1  (0,26) | 8,23  (0,28) | 8,38  (0,3) | 8,53  (0,33) |
|  |  | % used var. connectivity | 6,83  (1,88) | 21,27  (1,37) | 32,51  (1,49) | 41,54  (1,45) | 45,92  (1,35) | 50,87  (0,86) | 55,61  (0,66) | 58,36  (0,51) | 60,64  (0,32) | 62,71  (0,18) |
|  |  | % expl. var. age | 9,99  (4,45) | 30,32  (5,23) | 43,7  3(3,75) | 53,74  (3,71) | 64,32  (2,68) | 71,72  (2,3) | 78,7  (1,96) | 84,46  (1,46) | 89,16  (0,87) | 92,7  (0,64) |

**Supplementary Table 4.** Model performance SCFC. Model included region-specific connectivity values of structure and function across four different sample splits (60/40, 70/30, 80/20 or 90/10): RMSEP, % explained variance in age (sd) and % used variance of connectivity (sd) including up to ten components for real models or null models. For visualization see Supplementary Figure 2A.

| SC | | Component | 1 | **2** | 3 | 4 | 5 | 6 | 7 | 8 | 9 | 10 |
| --- | --- | --- | --- | --- | --- | --- | --- | --- | --- | --- | --- | --- |
| 60 | **Real**  **Model** | RMSEP | 6,23  (0,11) | 5,52  (0,11) | 5,36  (0,12) | 5,34  (0,14) | 5,52  (0,18) | 5,75  (0,21) | 6,08  (0,26) | 6,32  (0,3) | 6,55  (0,34) | 6,81  (0,38) |
|  |  | % used var. connectivity | 19,18(0,97) | 23,19  (0,37) | 25,13  (0,18) | 26,97  (0,16) | 28,4  (0,19) | 29,78  (0,16) | 30,78  (0,17) | 32,1  (0,27) | 33,34  (0,25) | 34,38  (0,23) |
|  |  | % expl. var. age | 19,28(1,78) | 45,91  (1,87) | 63,52  (1,7) | 73,25  (1,38) | 79,57  (1,33) | 84,22  (1,1) | 87,88  (0,99) | 90,19  (0,92) | 92,13  (0,78) | 93,76  (0,68) |
|  | **Null**  **Model** | RMSEP | 7,14  (0,36) | 7,54  (0,28) | 7,82  (0,27) | 8,07  (0,34) | 8,35  (0,38) | 8,67  (0,44) | 8,96  (0,48) | 9,26  (0,53) | 9,52  (0,58) | 9,75  (0,63) |
|  |  | % used var. connectivity | 13,3  (5,68) | 42,37  (5,14) | 48,32  (2,01) | 52,1  (0,44) | 55,37  (0,4) | 58,18  (0,34) | 60,56  (0,28) | 62,88  (0,25) | 65,44  (0,28) | 67,72  (0,27) |
|  |  | % expl. var. age | 11,57(7,71) | 30,92  (4,77) | 47,95  (3,25) | 59,09  (2,99) | 67,83  (2,38) | 74,76  (2,09) | 79,84  (1,87) | 84,02  (1,62) | 87,29  (1,42) | 89,78  (1,22) |
| 70 | **Real**  **Model** | RMSEP | 6,23  (0,09) | 5,51  (0,08) | 5,33  (0,09) | 5,29  (0,11) | 5,45  (0,14) | 5,69  (0,17) | 6,02  (0,2) | 6,28  (0,24) | 6,54  (0,27) | 6,85  (0,31) |
|  |  | % used var. connectivity | 19,21(0,79) | 23,16  (0,3) | 25,11  (0,15) | 26,9  (0,13) | 28,35  (0,16) | 29,65  (0,14) | 30,62  (0,17) | 31,93  (0,28) | 33,13  (0,24) | 34,17  (0,24) |
|  |  | % expl. var. age | 18,98(1,46) | 44,72  (1,55) | 61,29  (1,39) | 70,85  (1,21) | 76,73  (1,19) | 81,38  (1,04) | 84,97  (1,02) | 87,27  (0,99) | 89,31  (0,88) | 91,04  (0,78) |
|  | **Null**  **Model** | RMSEP | 7,11  (0,33) | 7,49  (0,25) | 7,78  (0,23) | 8,03  (0,29) | 8,3  (0,31) | 8,62  (0,36) | 8,92  (0,41) | 9,23  (0,45) | 9,52  (0,5) | 9,79  (0,55) |
|  |  | % used var. connectivity | 13,48(5,82) | 42,37(5,23) | 48,27(2,05) | 52,02(0,58) | 55,25(0,41) | 58(0,35) | 60,27(0,27) | 62,55(0,25) | 65,07(0,27) | 67,31(0,26) |
|  |  | % expl. var. age | 10,1  (7,23) | 27,73  (4,63) | 43,68  (3,04) | 54,22  (2,93) | 62,57  (2,41) | 69,34  (2,17) | 74,54  (2) | 78,87  (1,74) | 82,39  (1,6) | 85,18  (1,44) |
| 80 | **Real**  **Model** | RMSEP | 6,23  (0,07) | 5,49  (0,07) | 5,29  (0,07) | 5,23  (0,08) | 5,37  (0,1) | 5,6  (0,12) | 5,93  (0,15) | 6,18  (0,18) | 6,46  (0,21) | 6,78  (0,24) |
|  |  | % used var. connectivity | 19,24(0,59) | 23,15  (0,22) | 25,09  (0,11) | 26,85  (0,1) | 28,31  (0,13) | 29,57  (0,11) | 30,51  (0,15) | 31,89  (0,26) | 33,05  (0,23) | 34,06  (0,21) |
|  |  | % expl. var. age | 18,68(1,1) | 43,76  (1,24) | 59,66  (1,09) | 68,97  (0,99) | 74,4  (0,99) | 78,97  (0,92) | 82,39  (0,91) | 84,5  (0,92) | 86,55  (0,84) | 88,28  (0,81) |
|  | **Null**  **Model** | RMSEP | 7,06  (0,29) | 7,44  (0,23) | 7,72  (0,21) | 7,96  (0,26) | 8,23  (0,29) | 8,54  (0,32) | 8,84  (0,37) | 9,14  (0,41) | 9,44  (0,45) | 9,7  (0,49) |
|  |  | % used var. connectivity | 13,78(5,67) | 42,39  (5,08) | 48,24  (2,01) | 51,94  (0,45) | 55,16  (0,41) | 57,88  (0,31) | 60,07  (0,25) | 62,39  (0,24) | 64,94  (0,29) | 67,11  (0,27) |
|  |  | % expl. var. age | 8,7  (6,23) | 25  (4,12) | 39,97  (2,75) | 49,83  (2,82) | 57,96  (2,34) | 64,57  (2,24) | 69,62  (2,13) | 74  (1,93) | 77,53  (1,86) | 80,47  (1,7) |
| 90 | **Real**  **Model** | RMSEP | 6,22  (0,04) | 5,49  (0,04) | 5,26  (0,05) | 5,19  (0,06) | 5,32  (0,07) | 5,54  (0,08) | 5,86  (0,1) | 6,09  (0,12) | 6,38  (0,13) | 6,7  (0,16) |
|  |  | % used var. connectivity | 19,23(0,41) | 23,11  (0,15) | 25,05  (0,08) | 26,78  (0,07) | 28,27  (0,09) | 29,47  (0,08) | 30,4  (0,12) | 31,87  (0,21) | 32,98  (0,2) | 33,97  (0,15) |
|  |  | % expl. var. age | 18,49(0,76) | 43,0  2(0,84) | 58,26  (0,75) | 67,34  (0,68) | 72,35  (0,69) | 76,82  (0,66) | 80,02  (0,68) | 81,87  (0,7) | 83,87  (0,66) | 85,53  (0,64) |
|  | **Null**  **Model** | RMSEP | 7,07  (0,27) | 7,42  (0,21) | 7,69  (0,19) | 7,93  (0,22) | 8,18  (0,24) | 8,48  (0,28) | 8,77  (0,32) | 9,08  (0,35) | 9,36  (0,39) | 9,64  (0,43) |
|  |  | % used var. connectivity | 13,23(5,69) | 42,34  (5,23) | 48,17  (1,7) | 51,83  (0,7) | 55,05  (0,42) | 57,75  (0,32) | 59,87  (0,27) | 62,27  (0,24) | 64,86  (0,28) | 66,96  (0,26) |
|  |  | % expl. var. age | 8,47  (5,98) | 22,82  (3,87) | 36,98  (2,68) | 46,21  (2,67) | 53,87  (2,22) | 60,16  (2,18) | 65,03  (2,11) | 69,37  (1,99) | 72,84  (1,96) | 75,83  (1,85) |

**Supplementary Table 5.** Model performance SC. Model included region-specific structural connectivity values across four different sample splits (60/40, 70/30, 80/20 or 90/10): RMSEP, % explained variance in age (sd) and % used variance of connectivity (sd) including up to ten components for real models or null models. For visualization see Supplementary Figure 2B.

| FC | | Component | 1 | **2** | 3 | 4 | 5 | 6 | 7 | 8 | 9 | 10 |
| --- | --- | --- | --- | --- | --- | --- | --- | --- | --- | --- | --- | --- |
| 60 | **Real**  **Model** | RMSEP | 6,6  (0,12) | 6,61  (0,13) | 6,55  (0,15) | 6,57  (0,17) | 6,66  (0,2) | 6,84  (0,22) | 6,92  (0,24) | 7,1  (0,27) | 7,21  (0,29) | 7,32  (0,31) |
|  |  | % used var. connectivity | 11,5  (1,45) | 20,57  (1,64) | 25,28  (1,4) | 28,17  (0,96) | 31  (0,62) | 32,85  (0,66) | 34,81  (0,46) | 36,09  (0,35) | 37,29  (0,26) | 38,34  (0,2) |
|  |  | % expl. var. age | 11,64(1,49) | 24,78  (2,82) | 43,93  (2,12) | 58,04  (3,38) | 68,41  (1,8) | 77,73  (2,18) | 83,55  (1,34) | 88,5  (1,03) | 91,97  (0,81) | 94,53  (0,57) |
|  | **Null**  **Model** | RMSEP | 7,11  (0,3) | 7,48  (0,29) | 7,65  (0,28) | 7,78  (0,3) | 7,89  (0,31) | 8,02  (0,33) | 8,18  (0,36) | 8,35  (0,39) | 8,51  (0,42) | 8,63  (0,45) |
|  |  | % used var. connectivity | 9,99  (3,37) | 32,07  (2,54) | 45,86  (2,31) | 53,46  (1,72) | 59,17  (1,19) | 63,85  (0,83) | 67,66  (0,66) | 70,9  (0,4) | 73,38  (0,22) | 75,63  (0,19) |
|  |  | % expl. var. age | 9,94  (5,08) | 27,47  (5,86) | 42,44  (4,46) | 55,03  (4,09) | 65,65  (2,98) | 74,21  (2,62) | 81,44  (1,76) | 87,14  (1,22) | 91,28  (0,96) | 94,04  (0,67) |
| 70 | **Real**  **Model** | RMSEP | 6,62  (0,09) | 6,59  (0,09) | 6,51  (0,11) | 6,53  (0,13) | 6,62  (0,16) | 6,82  (0,18) | 6,91  (0,19) | 7,11  (0,23) | 7,23  (0,23) | 7,37  (0,26) |
|  |  | % used var. connectivity | 11,43(1,25) | 20,97  (1,3) | 25,3  (0,98) | 28,2  (0,78) | 31,01  (0,62) | 32,71  (0,6) | 34,65  (0,45) | 35,9  (0,35) | 37,12  (0,29) | 38,13  (0,21) |
|  |  | % expl. var. age | 10,97(1,24) | 22,21  (1,74) | 40,9  (1,68) | 54,23  (2,97) | 64,44  (1,56) | 74,31  (2,2) | 80,26  (1,35) | 85,53  (1,04) | 89,31  (0,85) | 92,31  (0,63) |
|  | **Null**  **Model** | RMSEP | 7,1  (0,28) | 7,43  (0,26) | 7,63  (0,25) | 7,76  (0,26) | 7,87  (0,27) | 8,01  (0,31) | 8,18  (0,32) | 8,37  (0,35) | 8,56  (0,39) | 8,71  (0,41) |
|  |  | % used var. connectivity | 9,79  (3,43) | 32,4  (2,51) | 46,27  (2,31) | 53,5  (1,88) | 59,21  (1,23) | 63,72  (0,84) | 67,36  (0,7) | 70,55  (0,39) | 73,01  (0,21) | 75,25  (0,18) |
|  |  | % expl. var. age | 9,02  (4,89) | 24,97  (5,66) | 39,23  (4,36) | 51,06  (3,94) | 61,45  (2,85) | 69,92  (2,56) | 77,44  (1,78) | 83,65  (1,33) | 88,32  (1,09) | 91,61  (0,82) |
| 80 | **Real**  **Model** | RMSEP | 6,62  (0,07) | 6,56  (0,07) | 6,47  (0,09) | 6,5  (0,1) | 6,57  (0,12) | 6,77  (0,15) | 6,87  (0,15) | 7,08  (0,18) | 7,21  (0,2) | 7,38  (0,22) |
|  |  | % used var. connectivity | 11,49(0,89) | 21,25  (0,94) | 25,27  (0,57) | 28,22  (0,68) | 31,02  (0,56) | 32,66  (0,54) | 34,58  (0,41) | 35,82  (0,33) | 37,03  (0,23) | 38  (0,18) |
|  |  | % expl. var. age | 10,37(0,87) | 20,38  (1,08) | 38,54  (1,29) | 51  (2,57) | 61,05  (1,18) | 71,01  (2,11) | 77,08  (1,21) | 82,48  (0,98) | 86,47  (0,82) | 89,84  (0,62) |
|  | **Null**  **Model** | RMSEP | 7,05  (0,23) | 7,37  (0,22) | 7,56  (0,22) | 7,7  (0,23) | 7,84  (0,25) | 7,98  (0,27) | 8,16  (0,29) | 8,37  (0,31) | 8,58  (0,35) | 8,75  (0,37) |
|  |  | % used var. connectivity | 9,85(3,33) | 32,74(2,45) | 46,52(2,32) | 53,48(1,76) | 59,23(1,13) | 63,67(0,78) | 67,23(0,56) | 70,39(0,36) | 72,85(0,25) | 75,03(0,17) |
|  |  | % expl. var. age | 7,87  (4,12) | 22,2  (5,03) | 35,21  (3,94) | 46,92  (3,8) | 57,41  (2,76) | 65,86  (2,62) | 73,62  (1,91) | 80,14  (1,38) | 85,19  (1,21) | 88,93  (0,94) |
| 90 | **Real**  **Model** | RMSEP | 6,61  (0,05) | 6,55  (0,05) | 6,44  (0,06) | 6,47  (0,07) | 6,53  (0,08) | 6,74  (0,11) | 6,84  (0,1) | 7,05  (0,13) | 7,19  (0,13) | 7,38  (0,15) |
|  |  | % used var. connectivity | 11,51(0,65) | 21,44  (0,72) | 25,23  (0,32) | 28,29  (0,52) | 31,01  (0,46) | 32,57  (0,47) | 34,5  (0,33) | 35,72  (0,28) | 36,94  (0,19) | 37,88  (0,17) |
|  |  | % expl. var. age | 9,97  (0,62) | 19,05  (0,66) | 36,64  (0,87) | 48,14  (1,87) | 58,12  (0,78) | 68,24  (1,87) | 74,22  (0,98) | 79,68  (0,81) | 83,77  (0,66) | 87,35  (0,49) |
|  | **Null**  **Model** | RMSEP | 7,01  (0,19) | 7,35  (0,21) | 7,54  (0,21) | 7,69  (0,21) | 7,84  (0,23) | 7,99  (0,24) | 8,17  (0,27) | 8,39  (0,29) | 8,61  (0,33) | 8,81  (0,36) |
|  |  | % used var. connectivity | 10,21(3,26) | 32,95  (2,4) | 46,67  (2,41) | 53,51  (1,64) | 59,3  (1,1) | 63,58  (0,78) | 67,07  (0,58) | 70,22  (0,4) | 72,66  (0,25) | 74,82  (0,17) |
|  |  | % expl. var. age | 6,73  (3,42) | 20,39  (4,71) | 32,67  (3,77) | 43,98  (3,72) | 54,04  (2,71) | 62,41  (2,72) | 70,09  (2,03) | 76,81  (1,5) | 82,03  (1,39) | 86,1  (1,08) |

**Supplementary Table 6.** Model performance FC. Model included region-specific functional connectivity values across four different sample splits (60/40, 70/30, 80/20 or 90/10): RMSEP, % explained variance in age (sd) and % used variance of connectivity (sd) including up to ten components for real models or null models. For visualization see Supplementary Figure 2C.


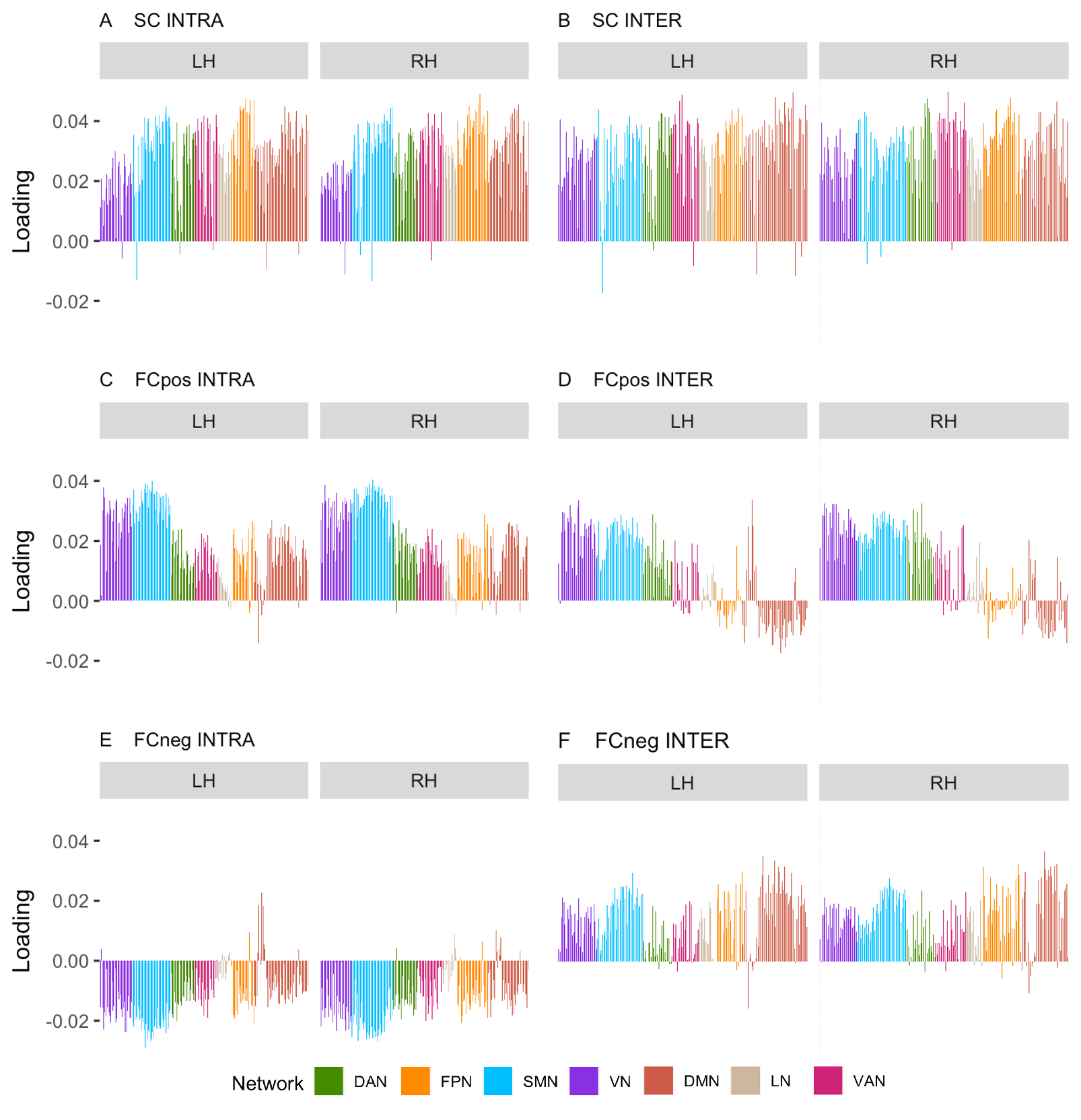


**Supplementary Figure 3.** 1^st^ components region-specific loading values for intra- (A, C, E) and inter-network (B, D, F) connectivity of SC (A, B), FCpos (C, D) and FCneg (E, F) visualized as bar plots (colored according to their respective network: violet = VN, blue = SMN, green = DAN, pink = VAN, grey = LN, orange = FPN, brown =DMN).


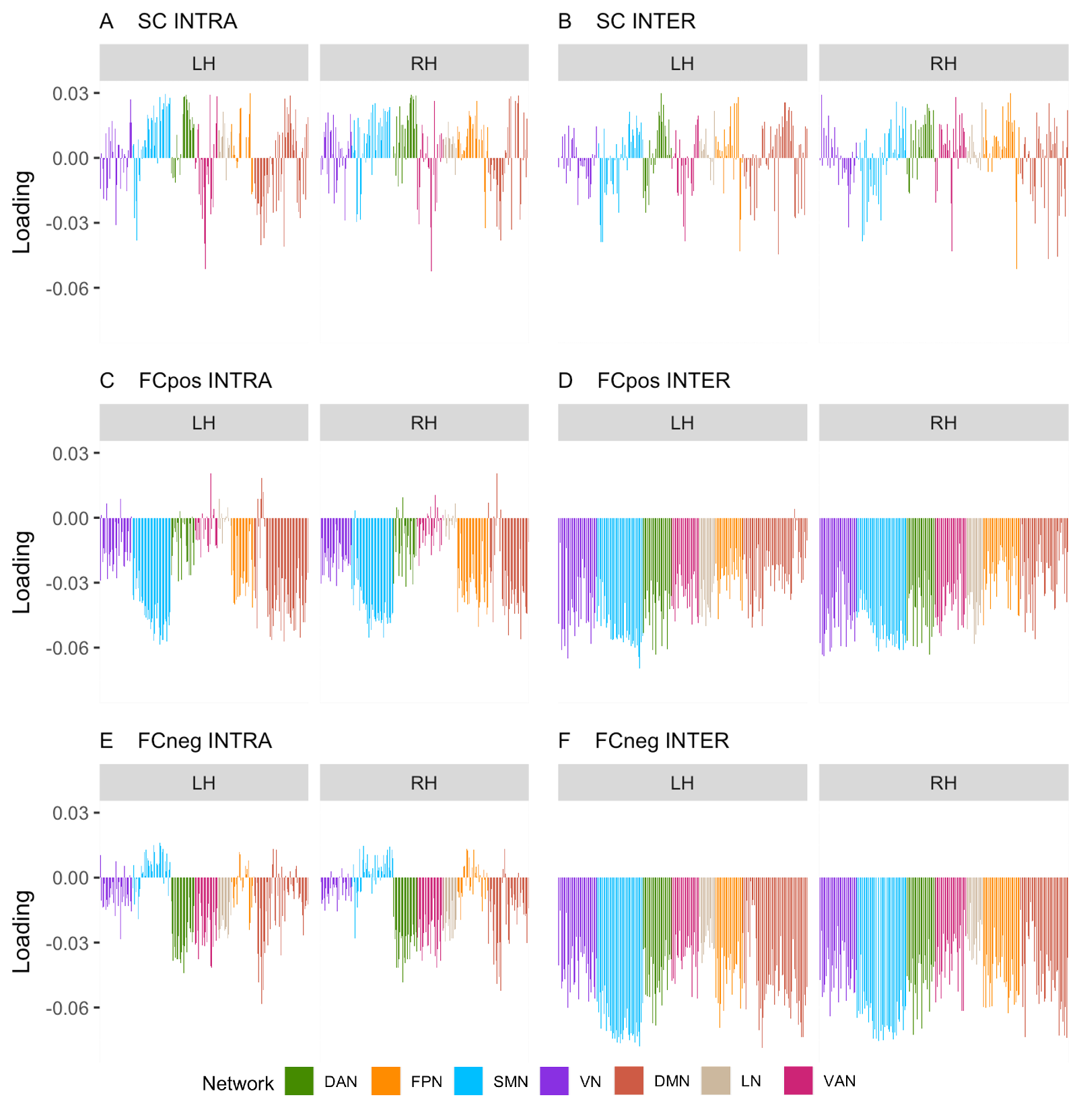


**Supplementary Figure 4.** 2^nd^ components region-specific loading values for intra- (A, C, E) and inter-network (B, D, F) connectivity of SC (A, B), FCpos (C, D) and FCneg (E, F) visualized as bar plots (colored according to their respective network: violet = VN, blue = SMN, green = DAN, pink = VAN, grey = LN, orange = FPN, brown =DMN).


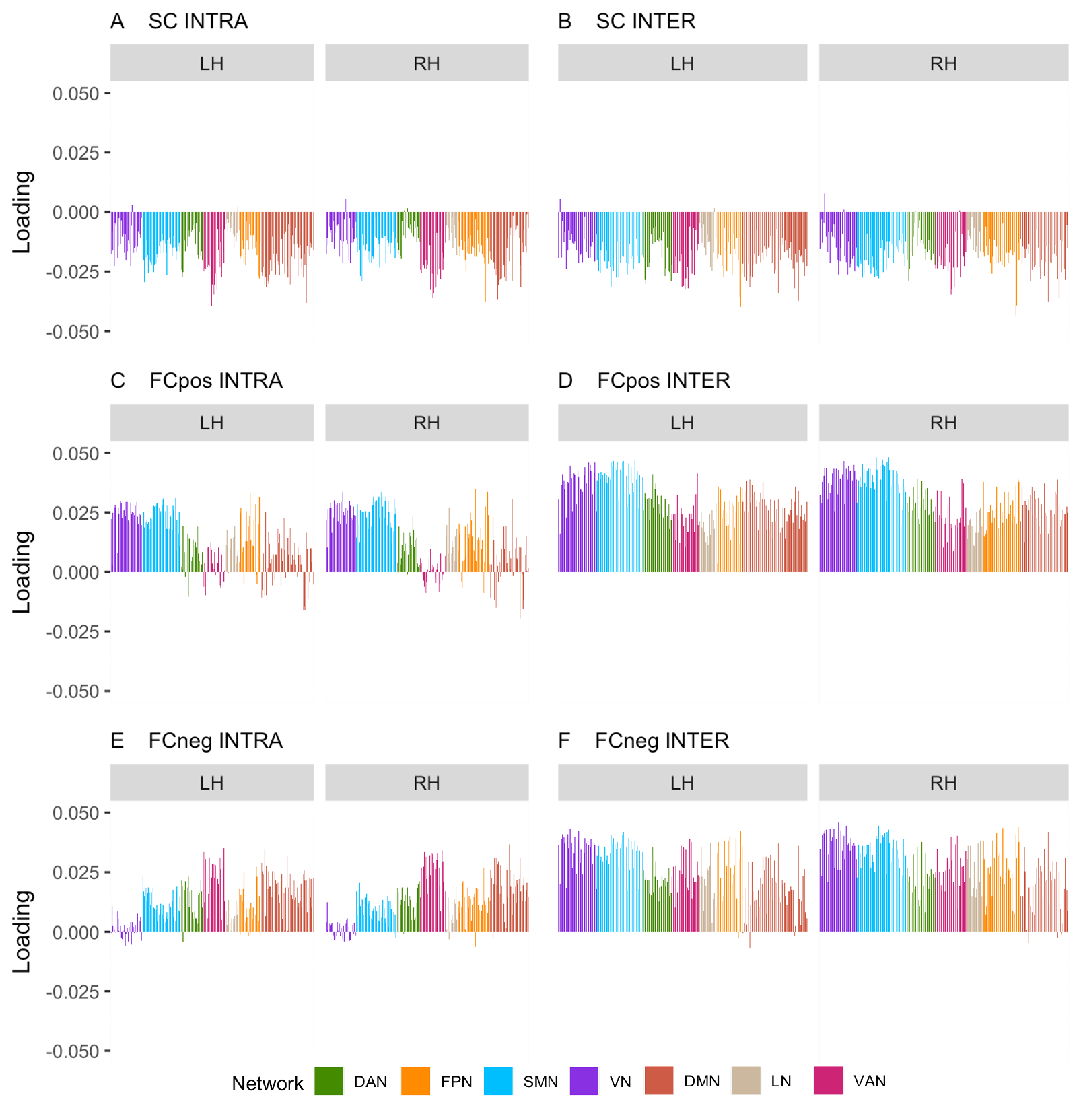


**Supplementary Figure 5.** 3^rd^ components region-specific loading values for intra- (A, C, E) and inter-network (B, D, F) connectivity of SC (A, B), FCpos (C, D) and FCneg (E, F) visualized as bar plots (colored according to their respective network: violet = VN, blue = SMN, green = DAN, pink = VAN, grey = LN, orange = FPN, brown =DMN).


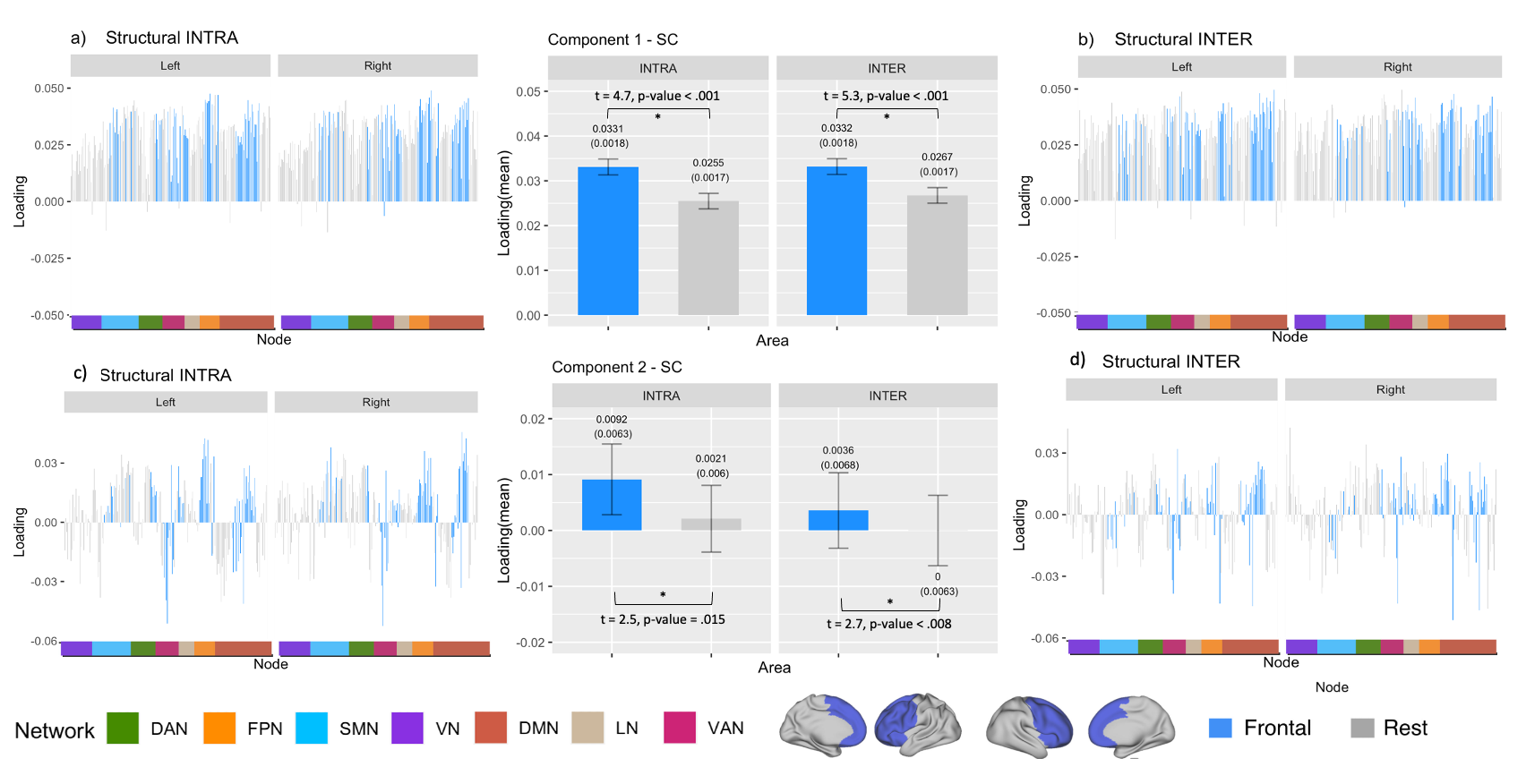


**Supplementary Figure 6:** For component 1 and component 2, comparisons of structural connectivity loading values within the frontal lobe (blue) as compared to the rest of the brain (grey). Node-wise loading values for intra- (a, b) and inter-network (c, d) are network-wise ordered (violet = VN, blue = SMN, green = DAN, pink = VAN, grey = LN, orange = FPN, brown =DMN) and colored blue as its location is within the frontal lobe. Additionally, mean loading values (standard deviation) across all regions within the frontal lobe or the rest of the brain are depicted for intra- (i, iii) and inter-network (ii, iv) structural connectivity and statistically compared (t-test: significant at p<.05, indicated by an asterisk).

**References**

Griffanti, L., Zamboni, G., Khan, A., Li, L., Bonifacio, G., Sundaresan, V., Schulz, U. G., Kuker, W., Battaglini, M., Rothwell, P. M., & Jenkinson, M. (2016). BIANCA (Brain Intensity AbNormality Classification Algorithm): A new tool for automated segmentation of white matter hyperintensities. *Neuroimage*, *141*, 191-205. <https://doi.org/10.1016/j.neuroimage.2016.07.018>
